# Transciptome profiling at early infection of *Elaeis guineensis* by *Ganoderma boninense* provides novel insights on fungal transition from biotrophic to necrotrophic phase

**DOI:** 10.1186/s12870-018-1594-9

**Published:** 2018-12-29

**Authors:** Mohammad Nazri Abdul Bahari, Nurshafika Mohd Sakeh, Siti Nor Akmar Abdullah, Redzyque Ramza Ramli, Saied Kadkhodaei

**Affiliations:** 1 Institute of Plantation Studies, Universiti Putra Malaysia, 43400 UPM, Serdang, Selangor Malaysia; 2 Faculty of Agriculture, Universiti Putra Malaysia, 43400 UPM, Serdang, Selangor Malaysia; 3 Research Institute for Biotechnology and Bioengineering, Isfahan University of Technology, Isfahan, 84156-83111 Iran

**Keywords:** Early defense, *Elaeis guineensis*, *Ganoderma boninense*, Necrotrophic, Pathogenesis-related protein, Transcription factor

## Abstract

**Background:**

Basal stem rot (BSR) caused by hemibiotroph *Ganoderma boninense* is a devastating disease resulting in a major loss to the oil palm industry. Since there is no physical symptom in oil palm at the early stage of *G. boninense* infection, characterisation of molecular defense responses in oil palm during early interaction with the fungus is of the utmost importance. Oil palm (*Elaeis guineensis*) seedlings were artificially infected with *G. boninense* inoculums and root samples were obtained following a time-course of 0, 3, 7, and 11 days-post-inoculation (d.p.i) for RNA sequencing (RNA-seq) and identification of differentially expressed genes (DEGs).

**Results:**

The host counter-attack was evidenced based on fungal hyphae and *Ganoderma* DNA observed at 3 d.p.i which became significantly reduced at 7 and 11 d.p.i. DEGs revealed upregulation of multifaceted defense related genes such as PR-protein (*EgPR-1*)*,* protease inhibitor (*EgBGIA*), PRR protein (*EgLYK3*) chitinase (*EgCht*) and expansin (*EgEXPB18*) at 3 d.p.i and 7 d.p.i which dropped at 11 d.p.i. Later stage involved highly expressed transcription factors *EgERF113* and *EgMYC2* as potential regulators of necrotrophic defense at 11 d.p.i. The reactive oxygen species (ROS) elicitor: peroxidase (*EgPER*) and NADPH oxidase (*EgRBOH*) were upregulated and maintained throughout the treatment period. Growth and nutrient distribution were probably compromised through suppression of auxin signalling and iron uptake genes.

**Conclusions:**

Based on the analysis of oil palm gene expression, it was deduced that the biotrophic phase of *Ganoderma* had possibly occurred at the early phase (3 until 7 d.p.i) before being challenged by the fungus via switching its lifestyle into the necrotrophic phase at later stage (11 d.p.i) and finally succumbed the host. Together, the findings suggest the dynamic defense process in oil palm and potential candidates that can serve as phase-specific biomarkers at the early stages of oil palm-*G. boninense* interaction.

**Electronic supplementary material:**

The online version of this article (10.1186/s12870-018-1594-9) contains supplementary material, which is available to authorized users.

## Background

Oil palm (*Elaeis guineensis* Jacq. *Dura x Pisifera*) is one of the main plantation crops in Malaysia and Indonesia and together these two countries contribute about 85 to 90% of global export [1, 2]. Palm oil which is recognised as one of the major sources of edible oil also serves as feedstock for oleochemicals and precursor for biodiesel fuel [3, 4]. The total export earnings from palm oil and palm oil products in Malaysia was reported at nearly USD18.5 billion [5]. Despite the huge export revenue from this commodity, oil palm plantation is facing major predicament due to basal stem rot (BSR) disease which hampers the oil palm production massively. BSR has been reported as a major threat in oil palm industry for over eight decades. It was estimated that in 2020, a total area of 443,430 ha or 65.6 millions of palm trees will be affected [6]. BSR is mainly caused by fungal infection on intact oil palm roots wherein the most prevalent species discovered was *Ganoderma boninense* [7–9]. BSR infects not only mature oil palm trees but also seedlings and younger plants where manifestation of the disease occurs earlier and more severe [8]. BSR is manifested by progressive decay of roots that disrupts water and nutrient transport to the upper part of mature oil palm trees which concomitantly will bring about frond wilting, yellowing of frond, un-opening of spear leaves and eventually resulting in stand collapse [10]. Regrettably, BSR-infected oil palms are symptomless during early stage of infection with the earliest symptom often observed on foliage when infection has progressed by 60–70% [11]. Once young oil palm plants show symptom of the disease they usually die within 1 or 2 years, while mature trees can survive for only another 3 or so years [12]. Thus, studies on early defense response are not just time and cost effective but provide insightful information on initiation of defense signaling networks upon recognition of pathogen.

*Ganoderma spp.* has been categorised as hemibiotrophs, with intermediate lifestyle of biotrophs and necrotrophs. Early stage of infection is the biotrophic phase whereby colonization of fungal on intact host plant cells takes place before initiating necrotrophic phase that involves extensive cell wall degradation [11]. Biotrophs survive by maintaining intact host cells for nutrient uptake, whilst necrotrophs involve killing of plant cells to infect and survive saprotrophically. Biotrophic infection is common during early interaction with pathogens whereby plant counteracts by enhancing production of reactive oxygen species (ROS) through an oxidative burst [13]. Consequently, plant executes programmed cell death (PCD) to restrict pathogen growth. This phenomenon is a form of hypersensitive response (HR) in which plant promotes cell death at and around the infection site [14]. Biotrophs utilize small amounts of cell wall degrading enzymes (CWDEs) to allow softening and loosening of cell wall without causing lethal effect to host cells [15]. Early defense response is also highly related to enhanced lignification of cell walls by plant to combat localized and controlled degradation of the cell wall by biotrophic fungi [16]. It is one of the strategies that plants employ to prevent penetration of pathogen’s toxins through cell wall degradation by CWDEs [17].

Regrettably, HR only induces transition of biotrophic to necrotrophic stage. Necrotrophs invade host tissues by extensively secreting CWDEs. The accessibility of CWDEs on cell wall is achieved by perception of necrotrophs to subvert host cell wall modification [18]. For instance, coactions of expansin and polygalacturonase which facilitate cell wall loosening were induced upon successful infection of necrotrophic pathogen *Botrytis cinerea* on *Solanum lycopersicum* [19]. Necrotroph also produces expansin-like protein to mediate penetration of hyphae [20]. Expansin-like protein provides protection for the hemibiotrophs, *Fusarium graminearum* from plant enzymatic degradation [21]. It has been postulated that biotrophic colonization is obligatory for hemibiotrophs to mediate successful infection while the time-period for switching from biotrophy to necrotrophy varies between pathogenic species [22]. Having intermediate lifestyle, hemibiotrophs may first overcome plant defense response during early colonization and subsequently deploy a more aggressive mode of attack for successful infection [23].

Studies on early interactions of plant-pathogens are crucial to allow screening for detection of potential threat of BSR especially on young palms. The present study attempts to investigate gene expression patterns in susceptible progeny of commercial oil palm (*Dura x Pisifera*) at early stages of *G. boninense* infection using high-throughput bioinformatics data (RNA-seq) via next generation sequencing (NGS) method. Despite the existence of a resistant variety (*Zaire* x *Cameroon*) [24], we decided to use the commercialized susceptible variety as it is vastly planted in oil palm plantations because this hybrid produces better yield performance compared to their parents [25, 26]. Previous study from our laboratory [27] using similar method and condition of treatment reported induced production of metabolites with anti-fungal properties in oil palm seedlings during early interaction (within a week of infection) with *G. boninense* suggesting activation of early defense responses in the host plant. Based on marker genes reported on biotrophs and necrotrophs, our study was able to differentiate the biotrophic stage before switching to necrotrophic phase which occurs later. The present work differs from previous reported studies which covered the later stages at three weeks post inoculation onwards [28, 29]. This work will enable a more complete understanding of oil palm defence response and is important for potentially early intervening strategies to protect the plant from severe infection.

## Results

### Preliminary screening of early defense response in *G. boninense*-infected oil palm roots

**Fig. 1:**
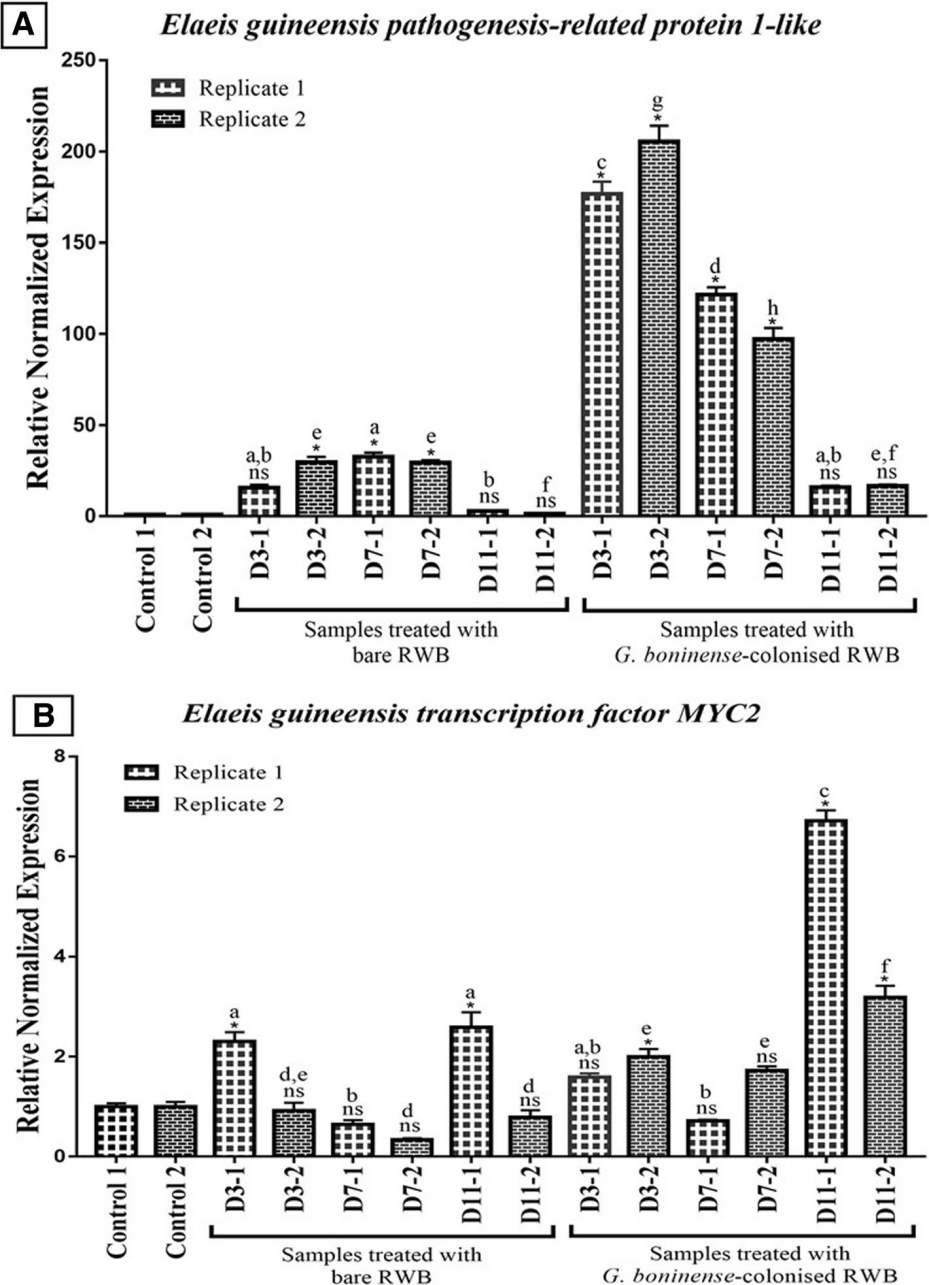
Preliminary screening of *EgPR1* and *EgMYC2* gene expression in *G. boninense*-infected oil palm roots. Histogram shows relative expressions of **a** *pathogenesis-related protein 1* (*EgPR1*) and **b** *transcription factor MYC2* (*EgMYC2*) genes at 3, 7, and 11 days-post-inoculation (d.p.i) compared to untreated control (**c**). The expressions of each gene were normalized by reference genes; *GAPDH 2, NADH 5* and *ß-actin* expression levels. Data are expressed as the mean ± SEM of three individual technical replicates of each sample. Preliminary screening by qPCR was carried out on control and treated (T0 and T1) samples in two biological replicates (1 and 2). Each replicate consisted of pooled root from six plants. * *P* < 0.01 is significantly differed compared to corresponding control as assessed by one-way ANOVA analysis followed by Tukey’s test. ns is not significant. Different superscript letters between samples (within replicate) indicate significant different (*P* < 0.01) in mean values. RWB: Rubber wood block

Eighty-four of 4-month-old oil palm seedlings were divided into two treatments which were inoculation with bare RWB (no fungal inoculum) as mock treatment (T0) and inoculation with RWB fully colonised with *G. boninense* (T1)*.* Artificial infection of oil palm seedlings with *G. boninense* was performed via sitting-technique to mimic the mode of *Ganoderma spp.* infection through root contact with fungal mycelia [27]. T0 and T1 samples were harvested at 3, 7 and 11 d.p.i. while untreated seedlings were used as control. Our preliminary screening via real-time quantitative PCR (qPCR) of transcriptional regulation in oil palm-*G. boninense* interaction showed two distinct phases of fungal attack suggesting early (3 d.p.i) and later (11 d.p.i) defense mechanisms (Fig. 1). The expression of pathogenesis-related protein 1 (*PR-1*) and transcription factor MYC2 (*MYC2*) genes, which are common genetic biomarkers for biotic stress were analysed [30–34]. *EgPR1* was highly expressed during the early phase of infection at 3 and 7 d.p.i before subsequently reduced at 11 d.p.i. Whereas *EgMYC2* showed highest gene expression during later phase of infection at 11 d.p.i. Based on the preliminary screening, we suggested that there are two plausible phases of defense response primed by oil palm expressing at very early (3 d.p.i) and later (11 d.p.i) interaction with *G. boninense*. Hence, the same batch of root samples harvested at the time points (3, 7 and 11 d.p.i) were used for further transcriptomic analysis through high throughput NGS.

### Scanning electron microscopy and PCR using *Ganoderma*-specific primers performed on artificially infected oil palm roots

**Fig. 2:**
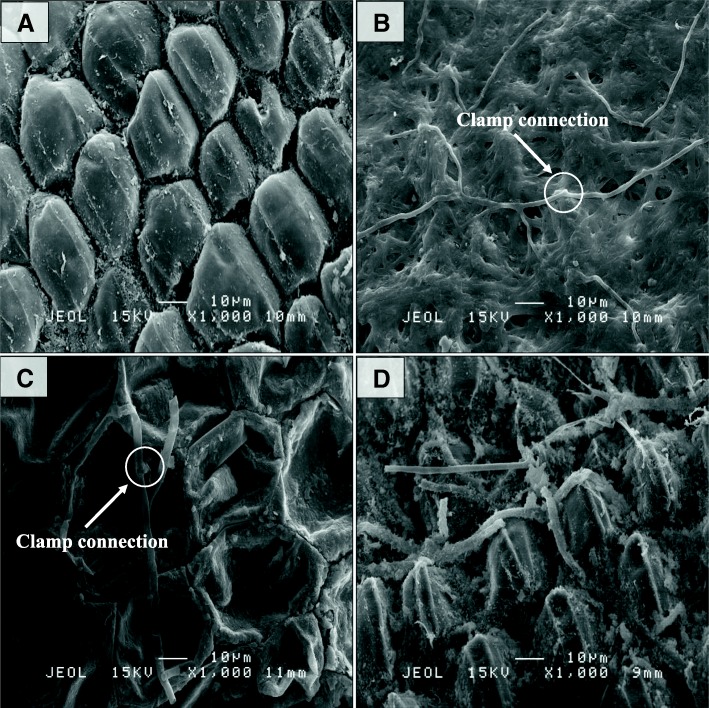
Scanning electron microscopy (SEM) of control and T1 oil palm roots with *Ganoderma boninense*. Root of samples were imaged at different days of post inoculation (d.p.i): (**a**) 0 d.p.i, (**b**) 3 d.p.i, (**c**) 7 d.p.i and (**d**) 11 d.p.i

Scanning electron microscopy on the outer layer of oil palm roots revealed differences in morphology between control and T1 samples. The superficial layer of control samples was intact and healthy, whilst the cell walls of *Ganoderma-*treated (T1 samples) were observed to be shrunken with uneven shape that showed symptoms of necrosis or apoptosis as early as 3 d.p.i (Fig. 2). The fungal hyphae network was undetected on control samples while surprisingly thick multilayers of fungal hyphae was present on the surface of 3 d.p.i roots but significantly reduced on root samples harvested at 7 and 11 d.p.i.

**Fig. 3:**
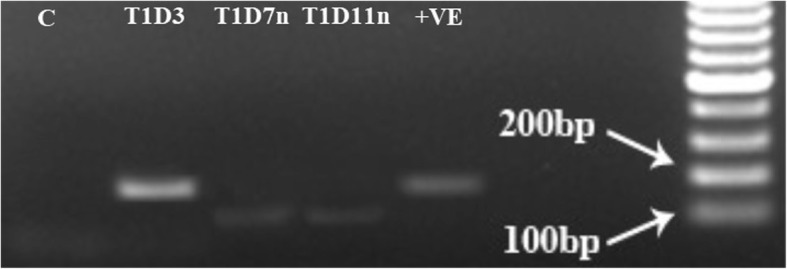
PCR amplification of *Ganoderma* species DNA using specific primer pairs of *Ganoderma* species. Lane C: uninoculated control; Lane T1D3: normal PCR for 3 d.p.i; Lane T1D7n: nested PCR for 7 d.p.i; lane T1D11n: nested PCR for 11 d.p.i; +VE: normal PCR for pure *Ganoderma* culture. The amplicon size for T1D3 and *+* VE are 223 bp whilst T1D7n and T1D11n amplicon size are 102 bp

The presence of *G. boninense* DNA in oil palm roots at 3, 7 and 11 d.p.i of T1 samples was further validated by PCR using *Ganoderma*-specific PCR primers. Primers of *G. boninense* DNA were retrieved from Genebank (accession number KM015454.1) from sequence of *G. boninense* strain PER71 internal transcribed spacer 1 (*ITS1*), partial sequence; 5.8S ribosomal RNA gene, complete sequence; and internal transcribed spacer 2 (*ITS2*), partial sequence with the expected product length of 223 bp size. The gel electrophoresis image in Fig. 3 shows concentrated and clear band on lane T1D3 (normal PCR for 3 d.p.i root sample). Bands of amplicons were not detected on normal PCR for 7 and 11 d.p.i. root samples. Hence, nested PCR was performed using both 7 and 11 d.p.i samples to validate any trace amount of *Ganoderma sp.* DNA. Nested primers were generated from sequenced PCR product of 3 d.p.i sample and the resulting amplicon length was decreased to 102 bp. Nested PCR (Fig. 3) resulted in faint bands for both root samples of 7 d.p.i (lane T1D7n) and 11 d.p.i (T1D11n). No band was detected from untreated control (lane C) sample. The alignment of original *G. boninense* ITS1/2 sequence with sequenced amplicon of normal and nested PCR showed conserved sequence which confirmed that *G. boninense* fungal hyphae was present in all T1 samples (Additional file 1). Furthermore, homology searches of the sequenced amplicon with biological sequences in GenBank matched only with *Ganoderma sp.* sequences with ≥95% identity. Consistent with the microscopic data, it was confirmed that *G. boninense* hyphae at 3 d.p.i was abundant whilst much reduced in 7 and 11 d.p.i.

**Fig. 4:**
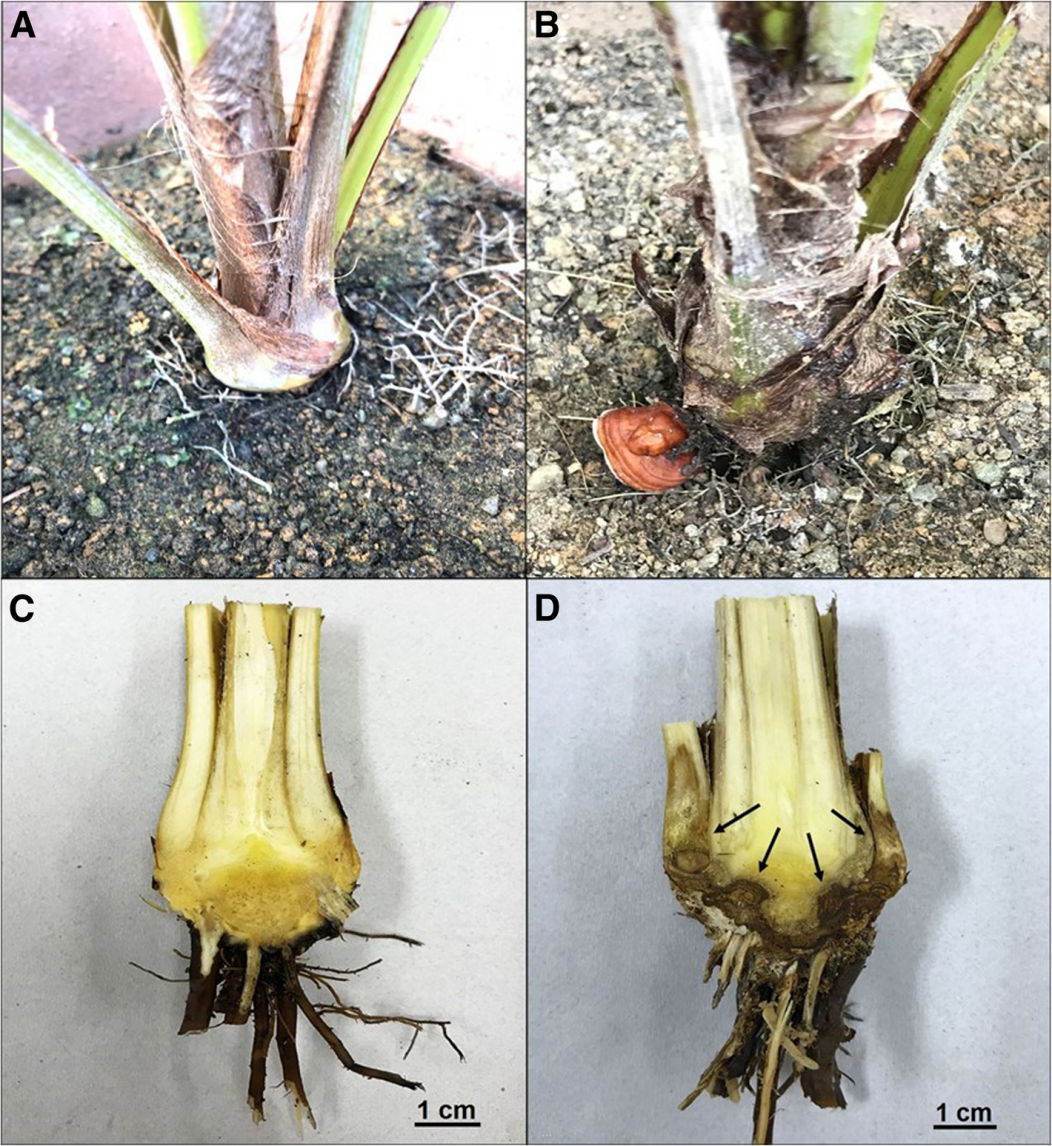
Signs and symptoms of *Ganoderma boninense* infection on oil palm seedlings. (**a**) healthy plant (uninfected) at 24 weeks after inoculation. (**b**) Appearance of *G. boninense* basidiomata on T1 oil palm stem base at 24 weeks after inoculation. (**c**) Stem base section of untreated control and (**d**) necrotic lesion (black arrows) in stem base of T1 oil palm at 24 weeks after inoculation

Observation on extended period of infection resulted in wilted leaves and emergence of fruiting body (basidiomata) at the bole of the T1 samples at the 24th week post inoculation, indicating a well-established necrotrophic infection (Fig. 4). An excised bole of the plant showed a decayed region indicating symptoms of necrosis while uninfected plant was healthy without *G. boninense* mass or necrotic lesions. Based on the observation of fungal hyphae on the outer layer of infected oil palm roots, the thick hyphae multilayers on 3 d.p.i which significantly dropped at 7 and 11 d.p.i raised possibilities that the fungus had been weakened by the plant. However, emergence of basidiomata at chronic infection proved that the plant finally succumbed to the disease, thus it shows that the drop of fungal hyphae at early interaction (within the treatment period) is not an indicator that the plant had overcome the fungal threats. We hypothesize that the switching to a more aggressive mode of attack by the fungus plays a critical role. Hence, we profiled transcriptomes of oil palm roots during interactions with *G. boninense* at 3, 7, and 11 d.p.i. to test the hypothesis.

### Transcriptomes of oil palm root during interactions with *G. boninense* at 3, 7, and 11 d.p.i

Biologically averaged samples are commonly practised in RNA-seq where pooled RNA samples, like in the present study from six oil palm seedlings were sequenced instead of individual samples. Biological averaging is not only cost efficient compared to mathematical averaging, but this method also could reduce the high biological variability which may be present among individual samples and raise the capability to detect differential gene expression between groups [35, 36].

**Fig. 5:**
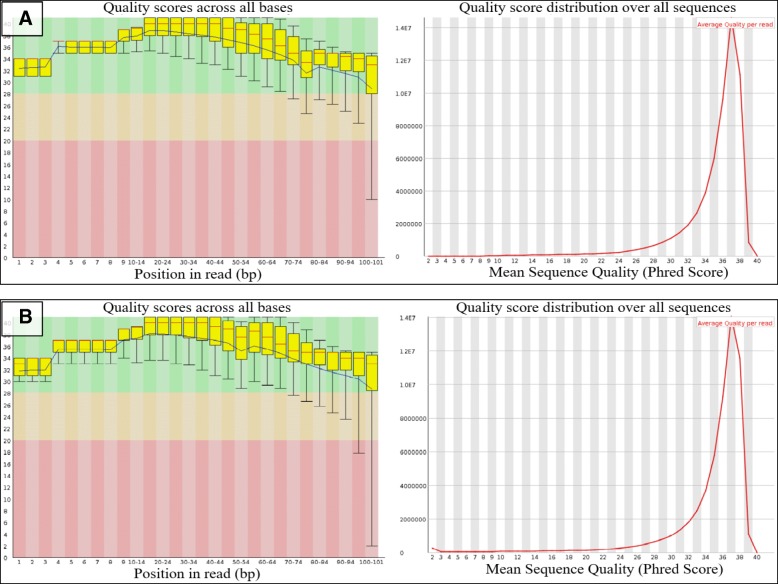
Per base sequence quality of samples generated by FASTQC. Yellow boxes demonstrated base-calling quality scores across all (**a**) replicate 1; and (**b**) replicate 2 sequencing reads

The RNA-seq generated 227,658,752 paired-end reads from the pooled two biological replicates of control, whilst the pooled biological replicates from 3, 7, and 11 d.p.i produced 227,400,216, 207,826,416 and 191,359,826 paired-end reads, respectively (Table 1) denoting that the pool of mRNAs in T1 samples decreased over time. Quality of the mRNA fragments from both biological replicates was measured using FastQC (Fig. 5a and b). An overview of quality values across all bases showed that the mean quality (blue line) for each base position lies in very good quality calls (green) region within the range of 28–38 quality scores. Furthermore, the quality score distribution graph showed that the highest number of sequences had mean sequence quality of 37. These paired-end reads were mapped to *E. guineensis* reference genome using Geneious for RNA-seq tool in the Geneious package. The percentages of mapped reads from samples were in the range of 54.14–60.21% with an average of 57.4%.

**Table 1:** Summary of NGS data of T1 samples

Replicate	Sample	Paired-end clean reads (fwd + rev)	Unmapped reads (%)	Mapped reads (%)	Contigs	GC (%)
1	Control	115,806,338	47,978,266 (41.50%)	67,828,072 (58.50%)	68,042,571	50.4
	3 d.p.i	116,036,532	48,074,890 (43.17%)	63,288,794 (56.83%)	63,473,939	50.3
	7 d.p.i	99,856,770	45,959,348 (46.03%)	62,010,298 (53.97%)	62,204,733	49.0
	11 d.p.i	96,334,214	39,143,968 (40.63%)	55,881,644 (59.37%)	56,049,226	49.9
2	Control	111,852,414	47,513,392 (42.48%)	64,339,022 (57.52%)	64,545,409	50.1
	3 d.p.i	111,363,684	50,771,616 (45.59%)	65,264,916 (54.41%)	65,460,036	49.6
	7 d.p.i	107,969,646	42,963,067 (39.79%)	56,893,703 (60.21%)	57,072,615	48.8
	11 d.p.i	95,025,612	39,734,017 (41.81%)	56,600,197 (58.19%)	56,777,437	49.3

Gene expression levels were calculated for each sample using transcript counts and presented in TPM unit [37]. Transcript counts is recommended in calculating the expression level of genes instead of reads or fragment counts since a single transcript can consists of multiple reads or fragments and yet they are not independent. Hence the accuracy of significance values is questionable. Normalization of transcript counts were performed using ‘Median of Gene Expression Ratios’ procedure which is identical to DESeq method [38].

**Fig. 6:**
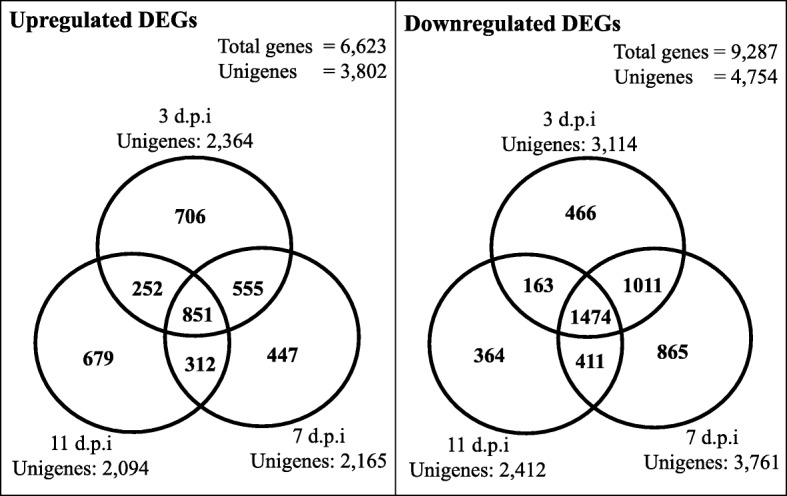
Venn Diagram of differentially-expressed genes in root of inoculated samples compared to uninoculated control samples. Genes were considered significantly upregulated or downregulated when their expression differences meet the cut-off values of fold change log_2_ ≥ |1.0| and *P*-value < 0.01

Difference in transcriptomic expression of individual sequences between control and T1 samples at different time frames was searched via comparing their transcripts expression level to identify DEGs. A given gene is considered as DEGs if its expression difference complies to the cut-off values of log_2_ FC ≥ |1.0| and *P*-value < 0.01. All DEGs were clustered into upregulated and downregulated based on positive and negative values of log_2_ FC respectively. As depicted in Fig. 6, the number of unigenes of downregulated genes (4754 DEGs) was 1.25-fold higher than upregulated genes (3802 DEGs). Among the upregulated genes, the highest number of DEGs was from 3 d.p.i. whereas the highest number of DEGs in downregulated genes was from 7 d.p.i. Based on observations for genes in common (overlapped region), both groups showed that the overlapping region between 3 d.p.i and 7 d.p.i had the highest number of DEG unigenes, followed by the overlapping between 7 d.p.i and 11 d.p.i. The lowest number of genes found in common was between 11 d.p.i and 3 d.p.i.

**Fig. 7:**
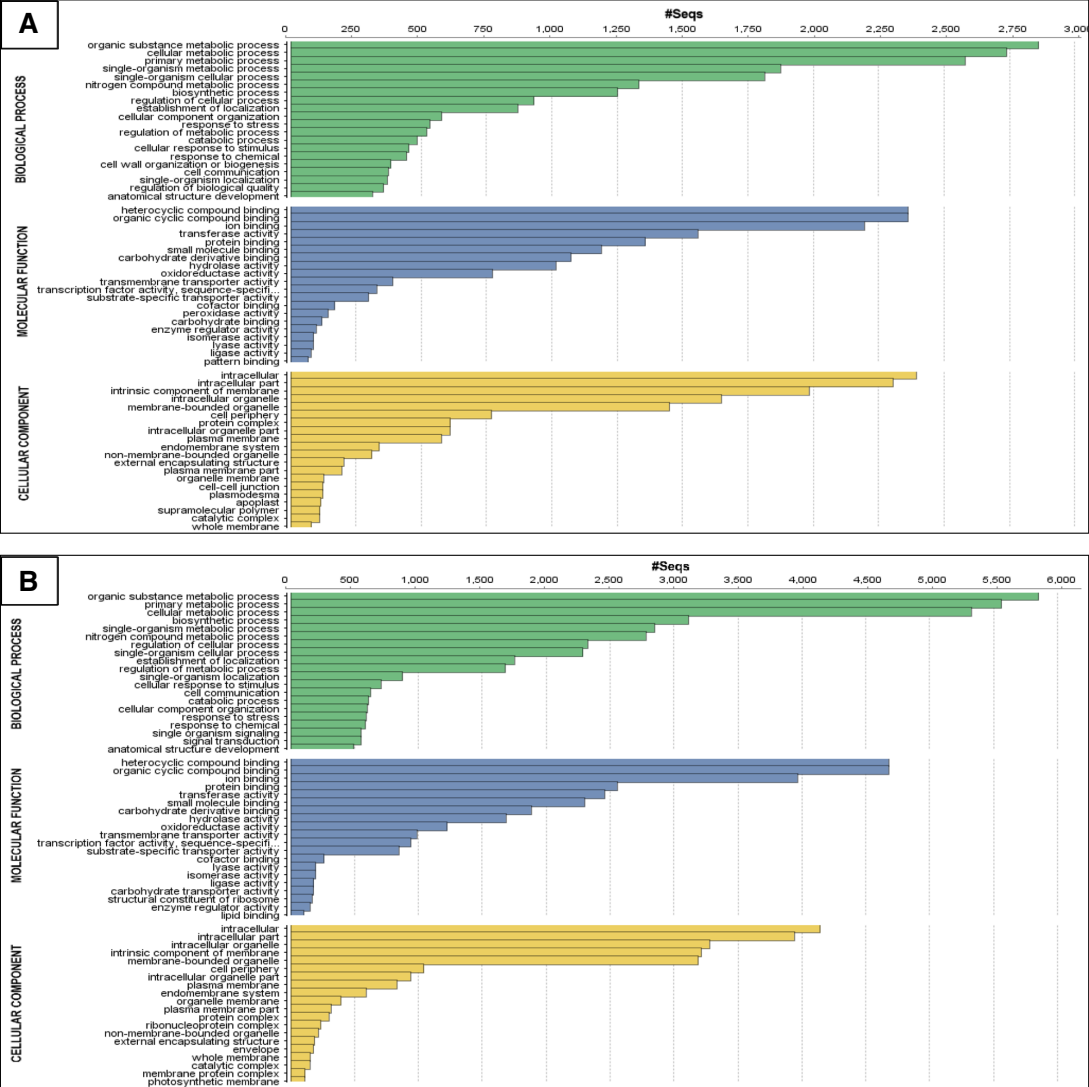
Gene Ontology (GO) functional categorization of differentially-expressed genes (DEGs). The bar charts represent top 20 GO distribution by number of sequences of (**a**) upregulated and (**b**) downregulated DEGs in T1 samples of oil palm roots during early interaction (3, 7, and 11 d.p.i) with *Ganoderma boninense* compared to untreated control

The upregulated and downregulated gene sequences were used to align with similar biological sequences using Basic Local Alignment Search Tool (BLAST) in database via the CloudBlast tools in Blast2GO. Top-hit species distribution revealed the best-aligned gene annotations of related plants with highest percentage of similarity and lowest e-value. With restriction to 20 blast hits and e-value cut-off of 1.0 × 10^− 3^, the most top-hit species was *Elaeis guineensis* with close to 9000 top-hits for upregulated genes and 15,000 top-hits for downregulated genes (Additional file 2). *Phoenix dactylifera* (date palm), and *Elaeis oleifera* (American oil palm) appeared as the second and third highest homolgy with maximum of ~ 150 top-hits. *P. dactylifera* is a close relative to *E. guineensis*, while *E. oleifera* is under the same genus *Elaeis* (tribe Cocoseae) in the family *Arecaceae*. The DEGs were annotated for Gene Ontology (GO) terms using Blast2Go Pro software. Fig. 7a and b show top 20 GO distribution (by level 3) by number of sequences of upregulated and downregulated DEGs respectively, which were categorized into biological process, molecular function, and cellular component. A single sequence could be present in more than one GO terms. Supplementary data showing statistics generated from blast and annotation method via Blast2Go Pro for quality control are available in Additional files 3 and 4 including annotation distribution, E-value distribution, sequence similarity distribution, and number of sequences with length.

The list of the top 20 GO of all categories was similar between upregulated and downregulated DEGs with exceptions for several terms. Furthermore, it was unsurprising to note that the number of downregulated DEGs were higher than the number of upregulated DEGs. However, ‘protein complex’, ‘non-membrane-bounded organelle’, and ‘external encapsulating structure’ from cellular component category have higher number of sequences in upregulated than downregulated DEGs. The majority of biological process GO terms either from upregulated or downregulated DEGs were related to various metabolic processes, while other possible defense-related GO terms include ‘response to stress’, ‘cell wall organization or biogenesis’, ‘biosynthetic process’, and ‘signal transduction’. Besides, molecular function GO terms exhibited multiple binding functions towards cyclic compounds, ions, proteins, enzymes, and metabolic substances. Different types of enzyme activities like transferase, hydrolase, oxidoreductase, lyase, isomerase, and ligase were also observed under this category. ‘Peroxidase activity’ from upregulated DEGs was not listed under downregulated DEGs for the top 20 GO terms, thus it showed that there was significant difference in the number of sequences from this enzyme function between upregulated and downregulated genes. The top 20 GO terms from cellular component category were related to intracellular parts, membranes and its components, spatially distinct organelles, ‘external encapsulating structure’, and ‘protein complex.

### Enriched GO terms of upregulated and downregulated DEGs at 3, 7, and 11 d.p.i

Subsequently, Gene Set Enrichment Analysis (GSEA) was performed using Blast2GO Pro to discover enriched GO terms in biological systems of oil palm during *G. boninense* colonization represented by DEGs. The *P*-values of differential gene expression between T1 and control samples were adopted as numerical values for each functionally annotated DEGs to create a ranked list for enrichment analysis with cut-off value of 0.01. GSEA was performed at 3, 7, and 11 d.p.i in order to deduce oil palm defense management strategies at different time intervals during early interaction with the hemibiotroph. Regardless of GO category, analysis on upregulated and downregulated DEGs at 3, 7, and 11 d.p.i showed 23, 27, and 33 (upregulated) while 94, 96 and 77 (downregulated) enriched GO terms, respectively. Significant changes in gene expression in the host plant were observed at different time intervals of early interactions. Despite susceptible-type of oil palm seedlings were used, enormous genes involved in defense-related processes were upregulated and downregulated during interaction with *G. boninense*. The selected enriched GO terms of upregulated and downregulated genes were listed in Table 2 and Table 3 respectively. Seventy-two hours (3 d.p.i) of interactions between the host and hemibiotroph revealed enriched GO terms involved in response to stress, hormone-mediated signaling pathway, auxin-signaling and cation binding. Response to stress was also enriched in upregulated genes at 7 d.p.i, beside other terms such as mitotic cell cycle process, O-acyltransferase activity, kinesin complex and cytoplasmic vesicle. Whereas after 11 days of inoculation, significant upregulation of genes involved in oxidation-reduction process, acyl transferase activity, movement of cell or subcellular component was observed. Besides the upregulated genes, the oil palm orchestrated significant attenuation of gene expression in response to fungal threat. In downregulated genes, ion transport, autophagy, signalling receptor activity and regulation of localization GO terms were enriched throughout the time points. Transcription factors activity for sequence-specific DNA binding and response to chemical were significantly affected at 7 and 11 d.p.i. Carbohydrate transport, oxidoreductase activity, organelle fusion, monooxygenase activity and symporter activity were enriched at 3 d.p.i. Catabolic process, vesicle-mediated transport, integral component of plasma membrane were enriched at 7 d.p.i. Whereas, regulation of metabolic process, aromatic compound biosynthetic process and developmental process were enriched at 11 d.p.i.

**Table 2:** Enriched GO terms of upregulated DEG unigenes of T1 samples compared to untreated control

3 d.p.i		7 d.p.i		11 d.p.i	
Biological Process					
GO:0006950	response to stress	GO:0006950	response to stress	GO:0055114	oxidation-reduction process
GO:0009755	hormone-mediated signaling pathway	GO:0030243	cellulose metabolic process	GO:0009888	tissue development
GO:0060918	auxin transport	GO:1903047	mitotic cell cycle process	GO:0006811	ion transport
GO:0010817	regulation of hormone levels	GO:0030154	cell differentiation	GO:0051704	multi-organism process
GO:0030001	metal ion transport	GO:0051704	multi-organism process	GO:0007010	cytoskeleton organization
		GO:0048646	anatomical structure formation involved in morphogenesis		
Molecular Function					
GO:0016651	Oxidoreductase activity, acting on NAD(P)H	GO:0046872	metal ion binding	GO:0046872	metal ion binding
GO:0010487	thermospermine synthase activity	GO:0008374	O-acyltransferase activity	GO:0043169	cation binding
GO:0048037	cofactor binding			GO:0010487	thermospermine synthase activity
GO:0043169	cation binding			GO:0050662	coenzyme binding
				GO:0016746	transferase activity, transferring acyl groups
				GO:0006928	movement of cell or subcellular component
Cellular Component					
GO:0005829	cytosol	GO:0005871	kinesin complex	GO:0044446	intracellular organelle part
		GO:0031410	cytoplasmic vesicle

**Table 3:** Enriched GO terms of downregulated DEG unigenes of T1 samples compared to untreated control

3 d.p.i		7 d.p.i		11 d.p.i	
Biological Process					
GO:0008643	carbohydrate transport	GO:0006811	ion transport	GO:0006811	ion transport
GO:0016491	oxidoreductase activity	GO:0005984	disaccharide metabolic process	GO:0019222	regulation of metabolic process
GO:0006914	autophagy	GO:0006914	autophagy	GO:0009059	macromolecule biosynthetic process
GO:0048284	organelle fusion	GO:0016192	vesicle-mediated transport	GO:0006914	autophagy
GO:0016071	mRNA metabolic process	GO:0008380	RNA splicing	GO:1902589	single-organism organelle organization
GO:0032879	regulation of localization	GO:0032879	regulation of localization	GO:0019438	aromatic compound biosynthetic process
GO:0006887	Exocytosis	GO:1902456	regulation of stomatal opening	GO:0032879	regulation of localization
GO:0006811	ion transport	GO:0042221	response to chemical	GO:0032502	developmental process
		GO:0009056	catabolic process	GO:0042221	response to chemical
		GO:0010817	regulation of hormone levels		
Molecular Function					
GO:0022804	active transmembrane transporter activity	GO:0038023	signaling receptor activity	GO:0010857	calcium-dependent protein kinase activity
GO:0004497	monooxygenase activity	GO:0003700	transcription factor activity, sequence-specific DNA binding	GO:0004872	receptor activity
GO:0038023	signaling receptor activity	GO:0005516	calmodulin binding	GO:0003700	transcription factor activity, sequence-specific DNA binding
		GO:0016705	oxidoreductase activity, acting on paired donors, with incorporation or reduction of molecular oxygen	GO:0051119	sugar transmembrane transporter activity
Cellular Component					
GO:0044459	plasma membrane part	GO:0005887	integral component of plasma membrane		

### Significant changes of gene expressions involved in defense response, cell wall modification, growth, and metabolism in the host plant

**Fig. 8:**
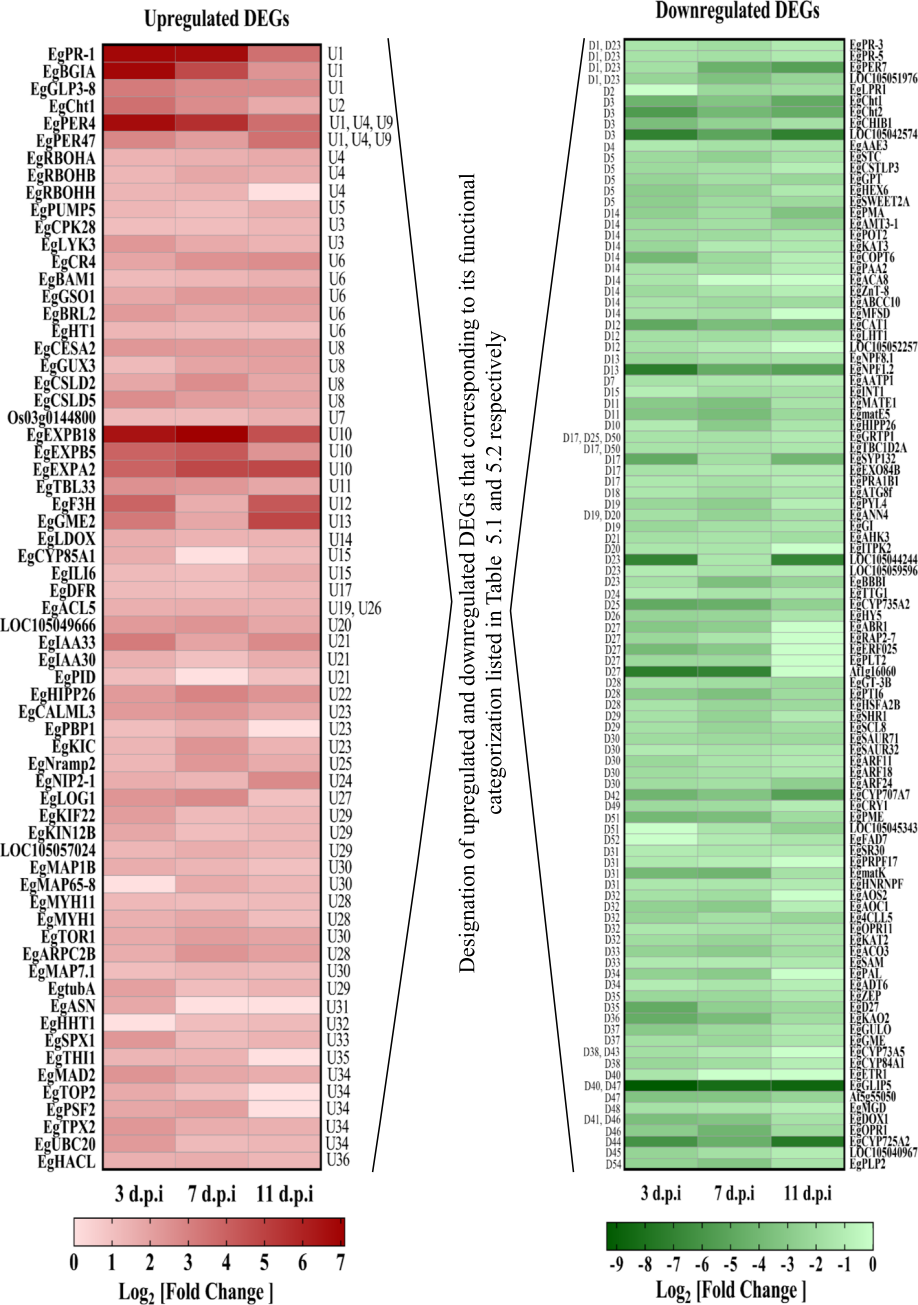
Expression pattern of selected upregulated and downregulated differentially-expressed genes (DEGs) of enriched GO terms. The colour intensity of each gene is based on Log_2_ [fold change] values of DEGs in T1 samples of oil palm roots during early interaction (3, 7, and 11 d.p.i) with *Ganoderma boninense* compared to untreated control

Analysis on differential expression of individual genes subset to the enriched GO terms has paved the way to observation of clusters of defense-related oil palm genes which either been activated or attenuated during interaction with *G. boninense*. Fold change of gene expression in T1 samples compared to control was applied to compute heatmap of selected significantly-expressed (*P*-value < 0.01) genes as shown in Fig. 8. Defense-related genes were the most affected by the host-fungus interaction whereby *pathogenesis-related protein 1-like* (*EgPR-1*), *Glu S.griseus protease inhibitor-like* (*EgBGIA*) and chitinases (*EgCht*) were significantly upregulated at 3 and 7 d.p.i and showed decreased in upregulation at 11 d.p.i compared to control. Other PR genes like *germin-like proteins* (*EgGLP*) and peroxidases (*EgPER*, an ROS scavenger) were both significantly upregulated and downregulated throughout the time points. Components of pattern-triggered immunity (PTI) signalling were shown to be significantly adjusted whereby *lysM domain receptor-like kinase 3* (*EgLYK3*), pattern recognition receptor (PRR) protein which is involved in perception of fungal-derived chitin molecule also known as pathogen- or damage-associated molecular patterns (PAMP or DAMP), was found to be both upregulated and downregulated, but upregulated genes were higher in term of fold-change compared to downregulated genes. Other receptor-like kinases (RLKs) or receptor-like proteins were involved either in surveillance of bacterial PAMP or related to growth, reproduction, differentiation and homeostasis processes. Another member of PTI signalling, *calcium-dependent protein kinase 28* (*EgCPK28*) was elevated at all time points.

As a mechanism to fortify the frontline barrier of defense in oil palm, genes associated with formation of primary and secondary cell wall and its modification were distinctively regulated in oil palm during interaction with *G. boninense*. The secondary cell wall biosynthetic genes, cellulose synthase A catalytic subunits (*EgCESA*) and cellulose synthase-like proteins (*EgCSL*) were only found in upregulated genes throughout all time points. Expansins are protein that regulate loosening and extension of cell wall. It was observed that both *expansin A-like* and *expansin B-like* were significantly upregulated in this study. Interestingly, *expansin-B18-like* (*EgEXPB18*) was outstandingly upregulated by 90- and 137-fold at 3 and 7 d.p.i respectively compared to the control. Expression of a gene involved in cutin, suberin, and wax biosynthesis, *omega-hydroxypalmitate O-feruloyl transferase-like* was elevated at later stage (7 and 11 d.p.i).

Several genes involved in lipid metabolism were significantly downregulated. A gene encoding a lipolytic enzyme, *GDSL esterase/lipases 5* (*EgGLIP5*) showed the highest fold downregulation (745-fold compared to control) at 3 d.p.i and only slightly decreased from 7 to 11 d.p.i. Monogalactosyldiacylglycerol synthase 1 (*EgMGD1*) that catalyzes the synthesis of a galactolipid, monogalactosyldiacylglycerol (MGDG) was significantly downregulated by 3-fold. Auxin cellular level, signalling and movement are critical for root and shoot architecture, organ patterning and tissue differentiation. In our data, auxin-responsive proteins (*EgIAA)*, repressor proteins in auxin signalling were upregulated whilst auxin response factor (*EgARF),* which mediates auxin-dependent transcriptional activation was downregulated at all time points. Furthermore, positive regulators of polar auxin efflux: *putative auxin efflux carrier protein* (*EgPIN*) and *protein kinase PINOID* (*EgPID*) were both upregulated thus may cause low cellular auxin level in the infected oil palm roots.

Genes involved in biosynthesis of secondary metabolites including flavonols, anthocyanidins, catechins and proanthocyanidins were upregulated but genes that confer biosynthesis of anthocyanins and fatty acid-derived secondary metabolites such as terpenes, terpenoids, and sphingolipids were downregulated. Furthermore, genes involved in biosynthesis of phytohormones like ethylene, jasmonate, L-ascorbate and gibberellin as well as their signalling pathway were downregulated at all time points. However, the downregulation was reduced at latter stage (11 d.p.i) for most of their biosynthetic genes such as jasmonate *allene oxide cyclase 1, chloroplastic-like* (*EgAOC1*) and *12-oxophytodienoate reductase 1-like* (*EgOPR1*); ethylene *1-aminocyclopropane-1-carboxylate oxidase-like (EgACO*); L-*ascorbate L-gulonolactone oxidase-like* (*EgGULO*); and ABA *zeaxanthin epoxidase, chloroplastic-like* (*EgZEP*).

Ion channels, multiple transport and carrier proteins for water, sugar, heavy-metal, drugs, ATP and ADP were downregulated throughout the experiments signifying transport of water and nutrients in oil palm was compromised during *G. boninense* attack. Nevertheless, a bHLH transcription factor *FER-LIKE IRON DEFICIENCY-INDUCED TRANSCRIPTION FACTOR* (*EgFIT*) was significantly upregulated at 11 d.p.i rather than 3 and 7 d.p.i. It is an integral regulator in response to iron deficiency which upon activation will trigger downstream iron uptake genes, like ferric reduction oxidase 2 (FRO2) and ATPase AHA2 [39]. In our RNA-seq data, *ferric reduction oxidase 2-like* (*EgFRO2*) was not expressed in control samples but was induced in T1 samples. Genes related to vesicle trafficking, autophagy, and pre-mRNA splicing activity were downregulated.

The expression pattern of DEGs was validated through qPCR using the same samples that have been sequenced for RNA-seq data. Several genes mentioned earlier particularly that played crucial roles in defense response were analyzed and demonstrated consistent expression pattern with RNA-seq data (Table 4). The expression profiles (up- and down-regulation between time-points) were largely identical. Besides, 5 out of 8 samples tested showed that all T0 samples were not affected by the treatment and their expression levels were significantly different compared to the corresponding T1 samples. This is crucial for minimizing the abiotic stress effect on the T1 samples.

**Table 4:** Validation of RNA-Seq data using

Genes	Fold change of expression of																	
	RNA-seq data						qPCR											
	in *Ganoderma boninense*-treated (T1 samples) compared to untreated control												in mock (T0 samples) compared to untreated control					
	Replicate 1			Replicate 2			Replicate 1			Replicate 2			Replicate 1			Replicate 2		
	3 d.p.i	7 d.p.i	11 d.p.i	3 d.p.i	7 d.p.i	11 d.p.i	3 d.p.i	7 d.p.i	11 d.p.i	3 d.p.i	7 d.p.i	11 d.p.i	3 d.p.i	7 d.p.i	11 d.p.i	3 d.p.i	7 d.p.i	11 d.p.i
*EgEXP18*	157.59*^a^	337.79*^b^	25.99*^c^	51.98*^d^	55.72*^d^	21.11*^e^	112.05 ± 2.68*^b^	105.28 ± 3.58*^b^	62.21 ± 2.44*^c^	119.44 ± 4.46*^d,e^	120.39 ± 4.70*^e^	10.36 ± 0.82^d^	13.31 ± 1.42^a^	11.08 ± 0.93^a^	3.40 ± 0.53^a^	14.97 ± 0.82^d,e^	6.76 ± 0.65^d^	2.88 ± 0.18^d^
*EgPG*	9.19*^a^	10.56*^a^	3.03^b^	6.50*^c^	4.92*^c^	ND	12.20 ± 1.22*^a^	11.68 ± 1.24*^a^	5.52 ± 0.79^a,b^	21.51 ± 2.19*^d^	18.76 ± 2.06*^d^	8.76 ± 1.27^c^	9.62 ± 1.53*^a^	2.52 ± 0.33^b^	2.31 ± 0.51^b^	8.67 ± 1.40^c^	2.43 ± 0.32^c^	2.25 ± 0.48^c^
*EgBGIA*	168.90*^a^	29.86*^b^	5.28^c^	78.79*^d^	24.25*^e^	ND	140.66 ± 30.61*^b^	29.46 ± 1.26^a^	3.90 ± 1.17^a^	147.81 ± 9.69*^f^	16.34 ± 3.73^d,e^	6.08 ± 0.66^d^	23.81 ± 3.56^a^	33.43 ± 2.21^a^	0.30 ± 0.03^a^	52.79 ± 5.05*^c^	43.20 ± 4.04*^c,e^	0.19 ± 0.05^d^
*EgCht1*	13.33*^a^	10.40*^a^	2.60*^b^	10.70*^c^	3.80*^d^	3.90*^d^	11.31 ± 0.44*^e^	4.84 ± 0.48*^a,b^	3.70 ± 0.08*^b,c^	27.26 ± 1.80*^j^	9.12 ± 0.31*^g,h^	4.79 ± 0.11^h,i^	5.62 ± 0.33*^a^	2.13 ± 0.09^c,d^	0.45 ± 0.02^d^	45.12 ± 1.69*^f^	11.00 ± 0.81*^g^	2.11 ± 0.16^i^
*EgERF113*	2.05*^a^	7.52^a^	13.30*^b^	3.65^**c**^	13.64^d^	45.30*^e^	6.30 ± 0.20*^b^	8.95 ± 0.50*^c^	21.04 ± 0.58*^d^	12.95 ± 0.85*^f^	7.35 ± 0.27*^f^	31.97 ± 2.26*^g^	0.20 ± 0.01^a^	0.06 ± 0.01^a^	0.13 ± 0.01^a^	1.37 ± 0.30^e^	0.79 ± 0.07^e^	0.70 ± 0.22^e^
*EgFIT*	5.50^a^	9.40*^a^	22.20*^b^	11.20*^c^	ND	76.50*^d^	8.40 ± 0.40*^b^	2.90 ± 0.12^a^	48.73 ± 2.12*^c^	4.72 ± 0.54*^e^	4.89 ± 0.34*^e^	18.32 ± 0.45*^f^	0.12 ± 0.02^a^	0.55 ± 0.03^a^	0.32 ± 0.05^a^	1.28 ± 0.13^d^	3.17 ± 0.13*^d,e^	2.55 ± 0.47^d^
*EgMTP10*	2.60^a^	2.50^a^	8.30^a^	5.60^b^	ND	35.50*^c^	8.18 ± 0.73^a^	7.81 ± 0.96^a^	172.68 ± 10.14*^b^	3.31 ± 0.56^c^	3.23 ± 2.71^c^	25.03 ± 1.56*^d^	ND	1.74 ± 0.10^a^	0.45 ± 0.02^a^	1.22 ± 0.08^c^	2.47 ± 0.07^c^	1.75 ± 0.22^c^
*EgPIN8*	2.30^**a**^	2.20^**a**^	3.00*^a^	7.80*^b^	5.40*^b^	41.80*^c^	75.66 ± 11.13^a^	7.44 ± 2.00^a^	1557.48 ± 33.63*^b^	27.48 ± 3.21^d,e^	57.97 ± 10.70*^e^	165.38 ± 14.24*^f^	ND	ND	ND	0.61 ± 0.05^**c**^	ND	ND

*EgEXP18: E. guineensis expansin-B18-like; EgPG: E. guineensis polygalacturonase-like; EgBGIA: E. guineensis glu S.griseus protease inhibitor-like; EgCht1: E. guineensis chitinase 1-like; EgERF113: E. guineensis ethylene-responsive transcription factor 113; EgMTP10: E. guineensis metal tolerance protein 10-like; EgPIN8: E. guineensis putative auxin efflux carrier component 8; EgFIT: E. guineensis FER-LIKE IRON DEFICIENCY-INDUCED TRANSCRIPTION FACTOR-like*

qPCREach replicate consisted of pooled root from six plants. Pairwise comparison of RNA-seq data was evaluated according to cut-off values of log_2_ fold change (FC) ≥ |1.0| and *P*-value < 0.01. Data of qPCR are expressed as fold change mean ± SEM of three individual technical replicates of T0 and T1 samples compared to untreated control. The fold expressions of each gene were normalized by three reference genes; *GAPDH 2, NADH 5* and *ß-actin* expression levels. Significant differences between qPCR groups were determined using one-way ANOVA analysis followed by Tukey’s test. * indicate significant different compared to corresponding control at: *P* < 0.01 and log_2_ FC ≥ |1.0| for RNA-seq; and *P* < 0.01 for qPCR. Different superscript letters (a-j) between samples (within replicate) indicate significant different at: *P* < 0.01 and log_2_ FC ≥ |1.0| for RNA-seq; and *P* < 0.01 in mean values for qPCR. ND: not detected

## Discussion

Cell wall modification and production of antimicrobial compounds in plants are the non-specific preformed defense responses which act as the first barrier against pathogen [40]. Induced defense system for PTI in plant is initiated with the detection of DAMP or PAMP such as fungal chitin by PRR of the host [41, 42]. However, pathogens are able to suppress PTI when it successfully delivers the effectors leading to effector-triggered susceptibility. At a later phase of resistance, the pathogen effectors thereafter perceived by nucleotide-binding site leucine-rich repeat (NB-LRR) for a more specific response the so-called effector-triggered immunity (ETI). ETI is an exaggerated version of PTI which could bring response over the resistance threshold level that lead to HR. Through time-course transcriptome analysis of oil palm seedlings artificially inoculated with *G. boninense*, the occurrence of these responses was monitored based on transcriptome profiling at the early stages of interaction which was within 11 d.p.i.

PRR proteins are involved in surveillance of pathogens attack through recognition of their signature-pattern molecules known as PAMPs. Among the PRR proteins, the lysM domain receptor kinase has been recognized to play a role in the fungal perception [43]. In the present study, *lysM domain receptor-like kinase 3* was significantly upregulated by 5-fold at 3 d.p.i with decrement at later time points. This will trigger PTI responses such as production of PR proteins, ROS and protease inhibitors during the biotrophic phase. In this study, the activation of PTI is further supported by upregulation of *EgCDPK28* which serves as a mediator for PTI responses.

RLKs are widely known for their roles in development, cells differentiation and perception of stimuli [44]. In this report, we presented upregulation of *CRINKLY-4* (*EgCR-4*) with ascending increment from 3 d.p.i until 11 d.p.i**.** CR-4 is one of the RLK mainly involved in roots stem cell differentiation and lateral roots formation [45]. CR-4 has been identified as one of several important extracellular domains of RLKs responsible in recognizing and perceiving diverse signals under both abiotic and biotic stresses [46]. It can be presumed that the stress signals are further transduced downstream to effector molecules via secondary signalling molecules, most commonly Ca^2+^ and ROS leading to orchestration of protein cascades to activate plant adaptation and/or defense responsive genes [47, 48].

PR-1 has been reported having prominent antifungal properties which combat fungal pathogens from further invading the host plant [49, 50]. Our discovery revealed significant upregulation of *EgPR-1* gene at 3 and 7 d.p.i. while significantly reduced at 11 d.p.i. Several PR-1 family members are synthesized in response to pathogen attack specifically as components for the local HR and systemic acquired resistance (SAR). Recent discovery by Gamir et al. showed that PR-1 binds and sequesters sterols from the membranes of microbes [51]. Sterol-auxotroph oomycete, *Phytophthora brassicae* is sensitive to PR-1, whereas sterol-prototroph pathogens are sensitive to PR-1 only when the production of sterol is interrupted. High dose of PR-1, particularly from within vacuole has the capability to sequester more sterols compared to their biosynthetic capacity, thus achieving the antimicrobial effect in vivo. In this study, abundant *G. boninense* hyphae network was observed at 3 d.p.i. We are proposing that reduction of the hyphae network at the later stages (7 and 11 d.p.i) could be due to sequestration of their ergosterol by the highly expressed PR-1 which left the fungus sterol-deficient. Experimental evidences from Choon and colleagues proved that ergosterol is produced by *G. boninense* as their primary metabolite in primary cell wall development [52]. Several studies have shown that *G. boninense* colonization and its growth phase can be determined by measuring the concentration of ergosterol [53, 54].

ROS is a unique molecule that serves both physiological and stress-related functions by playing the role as signalling molecules for redox homeostasis and PTI responses [55, 56]. Peroxidases are another well-known PR-protein belonging to PR-9 family that are induced in plant host during pathogen infection [50]. Peroxidases are expressed in higher plants under colonization of fungi [57] and other microbes to limit pathogen spread by providing structural barrier and creating an extremely unpleasant environment via heavy production of ROS and reactive nitrogen species (RNS) [58] at the cell wall matrix level that promote HR and SAR. Intriguingly, the transcripts of the peroxidase family of genes such as *EgPER3, EgPER4* and *EgPER47* were being highly upregulated in oil palm host during *Ganoderma* attack from 3 d.p.i until 11 d.p.i.

Besides, the significantly upregulated NADPH oxidase: respiratory burst oxidase homolog proteins (*EgRboHA* and *EgRboHB*) observed in the present study, could have assisted in the establishment of HR via the synthesis of apoplastic ROS [59]. The finding was supported by our previous study which demonstrated effective hyphae penetration of *Ganoderma spp.* and plant’s cell wall degradation as early as 24 h-post-inoculation indicating involvement of ROS and strategized degradation of cell wall during biotrophic stage [27]. Nevertheless, the high dose of ROS which is toxic to plant cells promotes susceptibility to necrotrophs [60].

Development of secondary cell walls (SCWs) is essential for various physiological processes in plant including growth, seed dispersal, pollen release and fertilization as well as defense response against pathogens attack [61]. SCWs consist of cellulose, lignins, hemicelluloses and some proteins to structurally support plant as well as regulate water transport [61, 62]. In the present study, cellulose synthase complexes which comprised of different isoforms of cellulose synthases (*EgCesAs*) responsible in SCWs biosynthesis were upregulated as early as 3 d.p.i. We are also reporting for the first time on the involvement of *Cobra-like 4* (*EgCOBL-4*) in defense response, a SCW biosynthetic gene which was upregulated at the later stage of infection against *G. boninense* (7 and 11 d.p.i). Arabidopsis COBL-4, ortholog of Brittle culm 1 has been reported to contribute in biogenesis of cellulose component as well as secondary cell wall thickening [63–65].

We also identified two out of four subfamilies of the transcripts for expansins (*EgEXPA* and *EgEXPB*) known to be responsible in cell wall expansion and loosening [66]. Expansins were mainly studied under abiotic stress due to water deficit [67–69]. Expansin has also been reported in cell wall alteration caused by flooding injury in soybean seedlings [70], thus it may be suggested that expansin was upregulated due to wounding by *Ganoderma* attack. A report has addressed regulation of expansin-like A2 against necrotrophic attack of *B. cinerea* on *A. thaliana* [71]. Our present data demonstrated 28-fold upregulation of *expansin A2-like* at 7 and 11 d.p.i. during *G. boninense* interaction. Two newly discovered expansins in the present study were *expansin B18* **(**upregulated 90 and 137-fold**)** as well as *expansin B5* (upregulated 15 and 19-fold) at 3 and 7 d.p.i. respectively. It was postulated that the ability of expansins to break noncovalent bonding of polysaccharides allow larger exposure of surface glucans of cellulose leading to cellulase enzymatic attack [72]. However, expansion and loosening of cell wall will increase susceptibility to necrotrophs infection.

During pathogenesis, pathogens secrete digestive proteases which facilitate degradation of plant proteins into smaller compounds beneficial as nutrient sources [73]. The proteolytic process is crucial for pathogen’s growth and cell proliferation within host cells. As pathogen proteases and their digested products are being administered, protease inhibitors (PIs) are released by host plant to inhibit the proteolytic enzyme as one of the resistance responses [74]. While PIs can be found naturally in plant to regulate many biological processes such as development [75] and abiotic stress induced-PCD [76], they are highly upregulated spatiotemporally during biotic stress [77–80]. PIs accumulate not only at the site of injury, but also at distal locations to prevent further protease digestive activities [74, 81]. Thus, protease inhibitors are recognized as one of the major inducible defenses to combat against phytopathogens [82, 83]. We are reporting for the first time a highly expressed *EgBGIA* from the less studied potato type 1 serine PIs family with 115-fold upregulation at 3 d.p.i. Interestingly, this PI was upregulated to the same level of PR-1 genes which is a prominent plant defense protein to combat fungal threats. Mostly produced by solanaceous plants, potato type 1 and II serine PIs have only been reported against herbivory attack [84], hence paving the way to further study on this gene, whether it has specific involvement in the responses against *Ganoderma* attack.

GDSL esterase/lipase is a lipolytic enzyme with conserved GDSL motif and wide substrate specificity. Arabidopsis GDSL lipase, *AtGLIP1* was reported to have a positive effect in conferring resistance towards *Alternaria brassicicola*, while its homolog *AtGLIP2* is involved in defense by inhibiting auxin response [85]. On the other hand, Gao and colleagues showed that rice infected with blast fungus *Magnaporte oryzae* treated with *OsGLIP1/2-RNAi* demonstrated reduced symptoms of disease, while *OsGLIP1/2*-overexpressed plant showed enhanced diseased symptoms. Thus, they proposed that *OsGLIP1* and *OsGLIP2* have negative regulatory role towards disease resistance in rice [86]. The dual positive and negative regulatory role indicates the diverse catalytic properties of GLIP1/2 in lipid metabolism. From our data, oil palm *EgGLIP5* expression was significantly reduced during early interaction with *G. boninense.* It was interesting to report on this gene, but further experimental verification is needed to ensure if *EgGLIP5* plays similar function as *OsGLIP1/2* in plant immunity. Gao et al. also reported that high level of monogalactosyldiacylglycerol (MGDG) corresponds to overexpression of *OsGLIP1* [86]. Although MGDG is abundant in leafy vegetables, varied total and relative contents are also observed in other plant parts [87]. We found that *monogalactosyldiacylglycerol synthase 1* (*EgMGD1*) expression was also downregulated. Exogenous application of MGDG facilitated growth of pathogen signifying its negative role in rice immune response [86], whilst *MGD1* is required as a positive regulator in *Arabidopsis* to induce SAR [88].

Due to its role as primary growth promoter, auxin or indole-acetic acid (IAA) has been shown to oppose the development of induced-resistance in plant against biotic and abiotic stresses while supporting disease manifestations in numerous plants. Auxin perception involves transport inhibitor response 1 (TIR1) and auxin signalling F-box protein 1, 2, and 3 (AFB1, AFB2, AFB3) as receptors which upon auxin signal will direct proteasomal degradation of Aux/IAA repressor proteins through ubiquitin ligase SKP-Cullin-F box, TRANSPORT INHIBITOR RESISTANT1/AUXIN SIGNALING F-BOX (SCF^TIR1/AFB^) complex and derepress ARF to regulate transcription of auxin-responsive proteins. Exogenous treatment of oligosaccharides on tobacco and *Arabidopsis* improved protection of these plants against *B. cinerea*, however early application with auxin restore their susceptibility [89, 90]. In our data, members of the Aux/IAA transcriptional repressors, auxin-responsive protein IAA33 and IAA30-like were upregulated at 3 d.p.i while the transcriptional activator *EgARF4, EgARF11, EgARF18* and *EgARF24* were downregulated at the same time point. This could suggest that auxin signalling pathway was inhibited throughout the treatment period which in turn compromised growth.

Apart from the inhibition of auxin signalling, our data also showed that polar auxin transport in root cells has been facilitated by the upregulation of *putative auxin efflux carrier component 8* (*EgPIN8*) and *protein kinase PINOID-like* (*EgPID*). Based on accumulating research evidences, the plasma membrane-localized PIN are critical auxin efflux carrier component, while PID positively regulate polar trafficking of PIN [91]. Studies showed that over expression of PIN or PID strongly inhibits root hair growth, while exogenous auxin feed or application of PIN or PID inhibitors restores the growth [91]. Thus, exaggerated efflux of auxin from roots by the actions of *EgPIN8* and *EgPID* may cause shortage of intracellular auxin which in turn suppressed auxin signalling and subsequently inhibited oil palm quaternary root growth during *G. boninense* interaction.

Gene expression of *EgERF113* was highly elevated by approximately 29-fold suggesting recognition of necrotrophic attack at 11 d.p.i synergistic with large increment by 17-fold of *EgPR-1*. The results support recent studies claiming highly upregulation of transcription factor ERF113 in plant defense response against necrotrophs which subsequently promote PR-1 proteins [92, 93]. Overexpression of transcription factor *ERF113* (*RAP26.*L) was reported to promote wound defense response triggered by jasmonate and ethylene [94]. The perception of JA-Ile induces interaction between its receptor CORONATINE INSENSITIVE 1 and JAZMONATE ZIM-DOMAIN proteins leads to the relieve of repression on MYC2 [95, 96], which explains upregulation of MYC2 at 11 d.p.i. MYC2 as well as MYC3 and MYC4 are essential in promoting accumulation of secondary metabolites during plant resistance against various pathogens [97]. MYCs are known as master regulator of JA expression under stress response but they differ in specificity depending on the spatiotemporal accumulation. Induced upregulation of *EgMYC2* in our study supports the reports suggesting that MYC2 mediates JA-responsive genes against necrotrophic attack [30] predominantly in roots while MYC3 and MYC4 expressed mainly in aerial tissues [98–100]. We summarized the proposed functional categorization based on differentially expressed unigenes enriched in oil palm seedling roots during early interactions with *G. boninense* at 3, 7 and 11 d.p.i (Tables 5 and 6).

**Table 5:** Proposed functional categorization of upregulated DEG unigenes at different time points compared to untreated control

3 d.p.i	7 d.p.i	11 d.p.i
[U1] Pathogenesis-related protein activity, [U2] defense against chitin-containing fungal pathogens	[U1] Pathogenesis-related protein activity, [U2] defense against chitin-containing fungal pathogens	[U1] Pathogenesis-related protein activity, [U2] defense against chitin-containing fungal pathogens
U3] Pattern recognition receptor activity and PAMP-triggered immunity (PTI) signalling	[U3] Pattern recognition receptor activity and PAMP-triggered immunity (PTI) signalling	[U3] Pattern recognition receptor activity and PAMP-triggered immunity (PTI) signalling
[U4] ROS production, [U5] scavenging activity	[U4] ROS production, [U5] scavenging activity	[U4] ROS production, [U5] scavenging activity
[U6] Signal transduction involve in growth, development, reproduction, and differentiation	[U6] Signal transduction involve in growth, development, reproduction, and differentiation	[U6] Signal transduction involve in growth, development, reproduction, and differentiation
Cell wall formation: [U7] Primary, [U8] Secondary	Cell wall formation: [U7] Primary, [U8] Secondary	Cell wall formation: [U7] Primary, [U8] Secondary
Cell wall modification: [U9] lignin degradation, [U10] loosening and extension, [U11] O-acetylation of cell wall polymers	Cell wall modification: [U9] lignin degradation, [U10] loosening and extension, [U11] O-acetylation of cell wall polymers	Cell wall modification: [U9] lignin degradation, [U10] loosening and extension, [U11] O-acetylation of cell wall polymers
Biosynthesis of secondary metabolites: [U12] flavonols, anthocyanidins, catechins and proanthocyanidins, [U13] ascorbate, [U14] anthocyanidins, [U15] brassinosteroid biosynthesis and signalling, [U17] flavonoid metabolism	Biosynthesis of secondary metabolites: [U12] flavonols, anthocyanidins, catechins and proanthocyanidins, [U13] ascorbate, [U14] anthocyanidins, [U16] brassinosteroid signalling, [U17] flavonoid metabolism	Biosynthesis of secondary metabolites: [U12] flavonols, anthocyanidins, catechins and proanthocyanidins, [U13] ascorbate, [U18] ubiquinone, other terpenoid-quinone, phenylpropanoids, [U15] brassinosteroid biosynthesis and signalling, [U17] flavonoid metabolism
[U19] Repression of early auxin response genes, [U20] auxin transport, [U21] regulation of auxin signalling	[U19] Repression of early auxin response genes, [U20] auxin transport, [U21] regulation of auxin signalling	[U19] Repression of early auxin response genes, [U20] auxin transport, [U21] regulation of auxin signalling
Binding protein and transport: [U22] heavy-metal, [U23] calcium, [U24] water, [U25] iron	Binding protein and transport: [U22] heavy-metal, [U23] calcium, [U24] water, [U25] iron	Binding protein and transport: [U22] heavy-metal, [U23] calcium, [U24] water, [U25] iron
[U26] Negative regulation in the proliferation of xylem vessels	[U26] Negative regulation in the proliferation of xylem vessels	[U26] Negative regulation in the proliferation of xylem vessels
[U27] Conversion of gibberellin and cytokinin from inactive form into bioactive form	[U27] Conversion of gibberellin and cytokinin from inactive form into bioactive form	
[U28] Cytoskeleton organization: [U29] Kinesin, [U30] microtubule	[U28] Cytoskeleton organization: [U29] Kinesin, [U30] microtubule	[U28] Cytoskeleton organization: [U29] Kinesin, [U30] microtubule
[U31] Nitrogen assimilation, distribution and remobilization within the plant	U33] Adaptation to phosphate starvation	[U32] Biosynthesis of cuticular wax and suberin
[U33] Adaptation to phosphate starvation	U34] Cell cycle process	[U33] Adaptation to phosphate starvation
U34] Cell cycle process	[U35] Protection from oxidative damage	U34] Cell cycle process
[U35] Protection from oxidative damage		
[U36] Fatty acid oxidation	[U36] Fatty acid oxidation	[U36] Fatty acid oxidation

**Table 6:** Proposed functional categorization of downregulated DEG unigenes at different time points compared to untreated control

3 d.p.i	7 d.p.i	11 d.p.i
[D1] ROS scavenging activity		[D1] ROS scavenging activity, [D2] oxidative stress response
[D3] Defense against chitin- and glucan-containing and [D4] oxalate-producing fungal pathogens	[D3] Defense against chitin- and glucan-containing and [D4] oxalate-producing fungal pathogens	[D3] Defense against chitin- and glucan-containing and [D4] oxalate-producing fungal pathogens
Transport: [D5] sugar, [D6] water, [D7] ATP, [D9] protein, [D10] heavy-metal, [D11] drug, [D12] amino acid, [D13] peptide, [D14] ion	Transport: [D12] amino acid, [D13] peptide, [D6] water, [D14] ion, [D5] sugar, [D7] ATP, [D11] drug, [D15] inositol, [D16] various	Transport: [D5] sugar, [D14] ion, [D10] heavy-metal, [D15] inositol, [D12] amino acid, [D7] ATP, [D11] drugs, [D6] water, [D13] peptide
[D17] Vesicle trafficking	[D17] Vesicle trafficking	[D17] Vesicle trafficking
[D18] Autophagy	[D18] Autophagy	[D18] Autophagy
[D19] Signal transduction: [D20] osmotic response, [D21] histidine kinase, [D22] phosphatidylinositol signalling	[D19] Signal transduction: [D21] histidine kinase, [D20] osmotic response, [D22] phosphatidylinositol signaling	[D19] Signal transduction: [D20] osmotic response, [D21] histidine kinase
[D23] Pathogenesis-related protein activity	[D23] Pathogenesis-related protein activity	[D23] Pathogenesis-related protein activity
Growth: [D24] trichome and root hair development, [D25] homeostasis	Growth: [D24] trichome and root hair development, [D25] homeostasis	Growth: [D24] trichome and root hair development, [D25] homeostasis
Transcription factor activity in regulating [D26] photomorphogenesis, [D27] ethylene-responsive genes, [D28] defense response, [D28a] biotic and abiotic stress response, [D29] growth and development	Transcription factor activity in regulating [D26] photomorphogenesis, [D27] ethylene-responsive genes, [D28] defense response, [D28a] biotic and abiotic stress response, [D29] growth and development	Transcription factor activity in regulating [D26] photomorphogenesis, [D28] defense response, [D28a] biotic and abiotic stress response, [D29] growth and development
[D30] Auxin responsive genes	[D30] Auxin responsive genes	[D30] Auxin responsive genes
[D31] Pre-mRNA splicing activity	[D31] Pre-mRNA splicing activity	[D31] Pre-mRNA splicing activity
Biosynthesis of [D32] jasmonate, [D33] ethylene, [D34] salicylate, [D35] ABA, [D36] gibberellin, [D37] L-ascorbate and [D38] phenylpropanoids. Phytohormones signalling pathway: [D39] jasmonate, [D40] ethylene, [D41] ABA	Biosynthesis of [D32] jasmonate, [D33] ethylene, [D34] salicylate, [D35] ABA, [D36] gibberellin, [D37] L-ascorbate and [D38] phenylpropanoids. Phytohormones signalling pathway: [D39] jasmonate, [D40] ethylene, [D41] ABA	Biosynthesis of [D32] jasmonate, [D33] ethylene, [D35] ABA, [D36] gibberellin and [D37] L-ascorbate. Phytohormones signalling pathway: [D40] ethylene, [D41] ABA
[D42] Oxidative degradation of abscisic acid	[D42] Oxidative degradation of abscisic acid	[D42] Oxidative degradation of abscisic acid
Biosynthesis of secondary metabolites: [D43] anthocyanins, tocopherols, terpenes, terpenoids, oxylipins and sphingolipids, [D44] taxols, [D45] pterostilbene	Biosynthesis of secondary metabolites: [D44] taxols, [D45] pterostilbene, [D46] oxylipins	Biosynthesis of secondary metabolites: [D44] taxols, [D46] oxylipins, [D45] pterostilbene
[D47] Lipid metabolism activity that confer negative regulation in resistance towards fungal pathogen	[D47] Lipid metabolism activity that confer negative regulation in resistance towards fungal pathogen	[D47] Lipid metabolism activity that confer negative regulation in resistance towards fungal pathogen
[D48] Biosynthesis of structural component of photosynthetic membrane	[D48] Biosynthesis of structural component of photosynthetic membrane	[D50] GTPase-activating protein for Rab family protein
[D49] Photoreceptor activity	[D49] Photoreceptor activity	
[D50] GTPase-activating protein for Rab family	[D50] GTPase-activating protein for Rab family	[D50] GTPase-activating protein for Rab family
[D51] Cell wall modification	[D51] Cell wall modification	[D51] Cell wall modification
	[D52] Biosynthesis of 16:3 and 18:3 fatty acids	[D52] Biosynthesis of 16:3 and 18:3 fatty acids
[D54] Non-specific lipolytic acyl hydrolase activity	[D54] Non-specific lipolytic acyl hydrolase activity	[D54] Non-specific lipolytic acyl hydrolase activity

The molecular and physiological evidences regarding transition from biotrophy to necrotrophy are still awaiting elucidation. This brings up the question on how long should the biotrophic phase be before the transition? Apparently, the biotrophic phase needs to keep progressing until the host defense is overwhelmed. In *M. oryzae*-rice and *C. graminicola*-maize pathosystems, the establishment of disease is favoured even though the fungi were not able to dampen the magnitude of defense at the early stages of interaction [23]. Hence, *C. graminicola* and *M. oryzae* presumably could endure the elevated host defense until the point when they change to the necrotrophic mode. Based on our observation in oil palm during *Ganoderma* attack, upregulation of important genes involved in defense responses such as PR-proteins (*EgPR-1*)*,* protease inhibitor (*EgBGIA*), PRR proteins (*EgLYK3*) and chitinases (*EgCht*) was observed at 3 and 7 d.p.i before dropping to insignificant level at 11 d.p.i, suggesting the occurrence of the biotrophic phase whereby multifaceted plant defense responses were deployed to counteract the *G. boninense* attack. The subsequent reduction in the defense response suggests switching to necrotrophic phase by the fungus which was essential for successful infection. The result agrees with report suggesting suppression of pathogen-responsive genes by transcription factor *MYC2* during necrotrophic attack [30]. Furthermore, significant upregulation of the *EgFIT* at later phase (11 d.p.i) and minor induction of *EgFRO2* could be another clue for necrotrophic phase that caused disturbance in iron uptake. EgFIT is a central transcription factor required in upregulation of iron deficiency responses in root of *Arabidopsis* [101] hence suggested iron deprivation at later phase of the infection in the oil palm root. Despite other pathosystems showing distinct transition period (i.e. *C. graminicola* only took 72 h post infection to begin necrotrophy on maize), development and spread of the fungi in the host plant may vary across different species and rely on the infection conditions.

Vargas et al. hypothesized that increasing pressure by plant defense responses during biotrophy has augmented pathogen to shift into necrotrophy [23]. Genes involved in ROS production: *EgPER* and *EgRBOH* were upregulated and maintained throughout the treatment period in the present study may cause overwhelming ROS accumulation thus underwent self-propagation causing cell damage which promotes necrotrophic infection denoting transition from biotrophy [102]. Besides, the loosening and expansion of oil palm cell wall by expansin may contribute to increase susceptibility to necrotrophs.

**Fig. 9:**
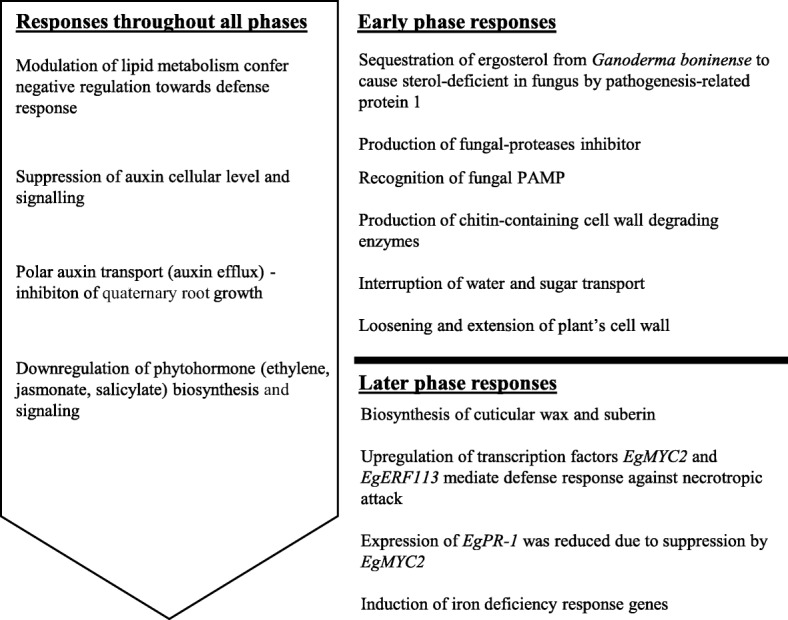
Proposed summary of defense-related events in oil palm roots during early interaction with *Ganoderma boninense.* Early phase responses (biotrophic phase) are the summary of events which occurred at 3 and 7 d.p.i while later stage responses (necrotrophic phase) are events occurring at 11 d.p.i based on analysis of DEGs

At the necrotrophic phase, we found out that CR-4 playing an important role in pathogen perception in oil pam. The oil palm then deploys another set of defense response against the necrotrophic attack that includes fortification of cell wall as well as rapid and significant upregulation of transcription factors. Transcription factor EgMYC2 is known to regulate defense response against necrotroph [30]. EgERF113 is proposed based on the present study as a novel transcription factor involved in biotic stress responses. The most commonly reported ERF transcription factors associated with biotic stress however, are ERF1 and ERF2 which are activated through ethylene and jasmonate signalling pathways [103–105]. It is evident that the oil palm finally succumbed to chronic infection. Schematic diagram on the proposed defense mechanism in oil palm during transition from the biotrophic to the necrotrophic phase is depicted in Fig. 9.

## Conclusions

Observations on early interaction between oil palm and *G. boninense* at different time points provided insights on early defense mechanism in oil palm to overcome the fungal threats even though the plant finally succumbed to BSR. Based on the evidences of the current study, several suggestions can be made: (1) the thick multilayer of *Ganoderma* hyphae observed at 3 d.p.i on oil palm root surface was significantly reduced at 7 and 11 d.p.i, indicating that the plant was likely to overcome the infection, however emergence of chronic-stage fruiting bodies indicated that the plant defense was overwhelmed by the fungus; (2) the fungus had possibly established a biotrophic relationship with the oil palm at early phase (3 and 7 d.p.i) of interaction as evidenced by significant upregulation of defense-related genes; and (3) the fungus may have switched its lifestyle to necrotrophy at later phase (11 d.p.i) of colonization whereby the elevated expression of the same defense-related genes was significantly reduced. The increasing pressure by plant defense responses during biotrophy could have triggered the transition as well as the overproduction of ROS which caused cellular damage and subsequent promotion of necrotrophic lifestyle to the fungus. The data provides evidence supporting the hemibiotrophic nature of this pathogen and it showed that practising hemibiotrophic routine is always an advantage for phytopathogen over the host. Analysis on DEGs revealed potential candidate genes to be further elucidated which can serve as phase-specific biomarkers at the early stages of oil palm-*Ganoderma* interaction.

## Methods

### Host plant and fungal inoculums preparation

*Ganoderma boninense* strain PER 71, an aggressive fungal pathogen causing BSR was obtained from Ganoderma and Diseases Research for Oil Palm (GanoDROP) Unit, Biology Division, Malaysian Palm Oil Board (MPOB) [106]. The fungus was isolated and purified from an infected oil palm in United Plantation Teluk Intan, Perak, Malaysia as described in Sundram et al. [107]. Four-month-old seedlings of susceptible oil palm (*Elaeis guineensis* Jacq. *Dura x Pisifera*), purchased from Sime Darby Plantation, Banting, Malaysia, were used as host plants. The seedlings were planted in Cobalt-60 (^60^Co) gamma radiation-sterilized (40 kGy) soil mix consisting of topsoil, peat and sand (3:2:1) placed in inert clay pot/vase and irrigated twice a day. Freshly prepared malt extract agar (Merck) was added onto sterile rubber wood block (RWB, 6 cm × 6 cm × 6 cm in dimension) and autoclaved at 121 °C for 30 mins, before inoculation with one-week-old *G. boninense* PER 71 cultured in potato dextrose agar (Difco). Inoculated RWBs were incubated at room temperature in the dark for 4 weeks to be fully colonised by the *Ganoderma* inoculum.

### Inoculation of *G. boninense* on oil palm seedlings (artificial infection)

A total of 84 of four-month-old oil palm seedlings (including control) were tested with two different treatments: inoculation with bare RWB (without fungal inoculum) as mock treatment (hereafter referred to as T0) and inoculation with RWB fully colonised with *G. boninense* (hereafter referred to as T1)*.* Throughout the treatment, all seedlings were arranged in a complete randomised design under conditionally-controlled plant house and watered twice daily using distilled water. Destructive sampling consisting of two biological replicates was performed at 3, 7 and 11 d.p.i (according to preliminary screening). Untreated seedlings were used as control. Each replicate consisted of pooled root samples from six randomly picked oil palm seedlings. The root samples were flash-frozen in liquid nitrogen and kept in − 80 °C until further use. Several untreated and T1 seedlings were kept for extended period for observation of chronic infection.

Artificial infection of *G. boninense* on oil palm seedlings were carried out using the method described by Idris et al. [106]. For inoculated samples, the colonised RWBs were placed in direct contact with the entire roots of the plant seedlings and were placed in clay pots which had been quarter-filled with the soil mixture. Soil was added until bole of the seedlings were fully covered.

For NGS, control and T1 samples were analysed for DEGs between different time points. DEGs of interest from the NGS data were validated using qPCR which also include the T0 samples for validation of *G. boninense* effect on oil palm gene expression.

### RNA extraction

Total RNA of all samples were extracted using the method described by [108] with minor modifications. Two grams of harvested root samples were ground to fine powder with mortar and pestle in liquid nitrogen. Six millilitre of RNA extraction buffer (50 mM Tris-HCl pH 9.0, 150 mM lithium chloride, 5 mM ethylenediaminetetraacetic acid pH 8.0, 5% (*w*/*v*) sodium dodecyl sulfate, 2 mM aurintricarboxylic acid) was freshly prepared and 0.4% of 2-mercaptoethanol was added into the buffer prior to use. Equal volume of phenol/chloroform (1:1) was added, and the mixture was centrifuged at 18,514 g for 30 mins at 25 °C. The aqueous phase was collected and transferred into a new tube. The addition of phenol/chloroform was repeated. Then, 6 mL of chloroform:isoamyl alcohol (24:1) was added and the tube was centrifuged at 18,514 g for 30 mins at 25 °C. The aqueous phase was collected and transferred into a new tube. Lithium chloride (8 M) was added to obtain a final concentration of 2 M and the mixture was kept overnight in 4 °C. After overnight incubation, the mixture was centrifuged at 12,857 g for 30 mins at 4 °C. The pellet was washed twice with molecular grade 90% ethanol and centrifuged at 12,857 g for 10 mins at 4 °C. The pellet was dried using Concentrator 5301 (Eppendorf, Germany). RNA pellet was dissolved in ultrapure nuclease-free water and kept in − 80 °C until further use. All centrifugations were performed using Centrifuge 5810R (Eppendorf, Germany).

### mRNA library construction and sequencing

Prior to mRNA library preparation, the RNA Integrity Number (RIN) of each sample was measured by Agilent 2100 Bioanalyzer (Agilent, USA) wherein only samples with RIN value of > 7.5 were accepted for sequencing. The mRNA library was constructed using the Illumina TruSeq RNA Library Prep Kit (Illumina, USA). Reads with an average length of 101 bp was used for sequencing on an Illumina HiSeq 2000 (Illumina, USA). Paired-end (2 × 100 bp) reads with an average length of 101 bp were sequenced by Illumina HiSeq 2000 system (Illumina, USA) at Macrogen, Korea. As pre-processing steps, the sequenced reads were saved in FASTQ format to determine the quality scores across all bases of short 101 bp paired-end reads using FastQC software.

### Genome assembly and identification of differentially expressed genes

The mRNA fragments were then mapped to *Elaeis guineensis* coding sequences as reference genome (retrieved from www.ebi.ac.uk/genomes) through Geneious software version 9.1.5 (Biomatters Ltd.). From align/assemble tools, Geneious for RNA-Seq was used as mapper with medium-low sensitivity using clean reads before mapping. Upon completion of the mapping step, the transcript abundance of each sample was calculated as transcript per kilobase million (TPM). To analyse the alteration in gene expression of infected oil palm compared to uninfected control, genes expressed from *G. boninense*-infected samples at each time point were compared to the genes expressed in absolute control sample. DEGs were evaluated according to stringent cut-off values of log_2_ fold change (FC) ≥ |1.0| and *P*-value < 0.01. DEGs that met the stringent cut off-values from comparative analysis between control and T1 samples of all time points were clustered according to upregulated or downregulated genes. The data of the sequenced mRNA have been deposited at European Nucleotide Archive under the accession number PRJEB27915.

### Scanning electron microscopy

Scanning electron microscopy was performed according to in-house method of Microscopy Unit, Institute of Bioscience, Universiti Putra Malaysia. Uninoculated and *G. boninense*-inoculated oil palm root seedlings were sliced into 1 cm^3^ using clean blades. Samples were fixed in 4% glutaraldehyde for 2 days at 4 °C and washed with 0.1 M sodium cacodylate buffer thrice for 30 mins each. Post-fixation was carried out in osmium tetroxide for 2 h at 4 °C, followed by dehydration through a graded acetone series (35, 50, 75, and 95% for 30 mins and 100% for 1 h, with three changes of acetone). The samples were then transferred into specimen vials and placed in critical dryer (LEICA EM CPD030) for about 30 mins. Samples were mounted onto stub and sputtered with colloidal silver and gold (BALTEC SC030) prior to viewing under a scanning electron microscopy (XL30 ESEM, Philips).

### GO and gene set enrichment analysis of DEGs

The DEGs were adopted for sequence homology searches (NCBI blast+) with biological sequences in CloudBlast database of Blast2GO with subset to Viridiplantae (taxa: 33090) [109]. Blastx program were executed in protein database, with limit to 20 blast hit results and restricted to maximum E-value of 0.001. Functional annotation and gene ontology of the DEGs were retrieved using default parameters in Blast2GO package and classified into biological process, molecular function and cellular component. Enrichment analysis of upregulated and downregulated GO terms was carried out via Gene Set Enrichment Analysis (GSEA) by applying the *P*-value of differential expression analysis as reference.

### DNA extraction from infected root tissue and pure culture of *G. boninense*

Mycelia of *G. boninense* PER 71 were streaked and inoculated into 150 mL freshly made potato dextrose broth (Difco) in 250 mL conical flasks. The cultures were incubated for a week using benchtop incubator shaker SI-600 (Lab Companion, Korea) at 37 °C with agitation at 150 rpm. The grown mycelia were rinsed using distilled water, filtered using filter papers and promptly grounded using mortar and pestle in N_2_ suspension. Powdered form mycelia were stored in − 80 °C and ready to be used for DNA extraction. DNA of *G. boninense* from 200 mg of mycelia and infected root tissues were extracted using Prescott and Martin, (1987) method [108] with minor modifications. DNA pellet of both *G. boninense* and all infected samples were dissolved in ultrapure nuclease-free water and stored in − 20 °C until further use.

### Validation of *G. boninense* presence within T1 oil palm seedlings via PCR and nested PCR

The DNA obtained from *G. boninense* PER 71 was used as control to validate the presence of infection within the roots of T1 samples. DNA of mycelia was amplified using primers of *G. boninense* strain PER71 internal transcribed spacer 1 (*ITS1*), partial sequence; 5.8S ribosomal RNA gene, complete sequence; and internal transcribed spacer 2 (*ITS2*), partial sequence with genebank accession number KM015454.1. Product length was expected to be 223 bp. Sequence of primer is listed in Table 7. Tubes containing reaction mixtures of DNA (5 ng), primers (5 ng each) and 2X KAPA Hifi HotStart Readymix (12.5 μL) were inserted in thermocycler (MyCycler™ Thermal Cycler System with Gradient Option, Bio-Rad, USA) with cycling parameters of 95 °C for 5 mins; 40 cycles of 94 °C for 35 s, 35 s at 63 °C, 40 s at 72 °C; and 72 °C for 10 mins. The amplicon of the PCR was sent for sequencing (Apical Scientific, Malaysia). Nested PCR was performed for 7 and 11 d.p.i root samples by using PCR product of 3 d.p.i root sample as template. Nested primer pair of the sequenced product was designed using Primer3 (v.0.4.0) based on certain criteria such as annealing temperature of primer pairs support separation of both PCRs based on the given parameter and high sensitivity of primers for improved detection threshold of nested PCR. Sequences of nested primers are listed in Table 7. The cycling parameters were similar to previous PCR with the exception of annealing temperature of 62 °C. Amplified products were dyed with 1.5 μL bromophenol blue dye and electrophoresed in 1% agarose gel. The gel was stained initially using FloroSafe DNA Stain and visualized using Gel Doc™ XR+ Imager (Bio-Rad, USA).

**Table 7:** Primers used for validation of *Ganoderma boninense* DNA of oil palm roots

Primer ID	Accession No.	Sense sequence (5′-3′)	Antisense sequence (5′-3′)
C (control)	KM015454.1	CAACGGATCTCTTGGCTCTC	GCCGATCAATAAAAGACCGA
T1D3 (3 d.p.i)	KM015454.1	CAACGGATCTCTTGGCTCTC	GCCGATCAATAAAAGACCGA
T1D7n (7 d.p.i)	–	GATCGGCTCCTCTCAAATGC	CGGTTAGAAGCTCGCCAAAC
T1D11n (11 d.p.i)	–	GATCGGCTCCTCTCAAATGC	CGGTTAGAAGCTCGCCAAAC
+VE	–	GATCGGCTCCTCTCAAATGC	CGGTTAGAAGCTCGCCAAAC

Note: C - primer for nested PCR from untreated control sample

T1D3 - primer for normal PCR from 3 day-post-inoculation sample

T1D7n* - primer for nested PCR from 7 day-post-inoculation sample

T1D11n* - primer for nested PCR from 11 day-post-inoculation sample

+ve -primer for normal PCR from *Ganoderma boninense* PER71 pure culture

* retrieved from sequenced PCR amplicon of T1D3 sample

### Experimental validation with qPCR

All primers encoding *EgPR-1*, *EgEXP18*, *EgPG*, *EgMYC2*, *EgBGIA*, *EgMTP10*, *EgCht1*, *EgERF113, EgPIN8* and *EgFIT* (Table 8) were designed using Primer3 (v.0.4.0) for qPCR. Preliminary screening of defense-related genes and validation of genes expression were performed using qPCR Green Master Mix LRox (2x) according to manufacturer’s protocol (Biotechrabbit GmbH, Germany). The qPCR cycling parameters were set as follow; 1 cycle of 95 °C for 3 mins, 40 cycles of 95 °C for 15 s and 62 °C for 30 s, followed by melt curve at 65 °C to 95 °C (5 s for every increment of 0.5 °C). Analysis of the qPCR were performed using Bio-Rad CFX Manager (Bio-Rad, USA). The specificity of each primer pair was verified by melt curve analysis. Stability of five endogenous controls (*Ubiquitin, Manganese Superoxide dismutase (MSD), GAPDH 2*, *β-actin* and *NADH 5*) was tested over all samples (control, mock and treated). Expression levels of all analyzed genes were normalized against the expression level of three most stable reference genes which were *GAPDH 2*, *β-actin* and *NADH 5*. Using more than one reference genes as normalization factor for qPCR data is needed to avoid the drawbacks of single gene normalization error [110]. Primers for *NADH5* and *β-actin* were designed based on Kwan et al. [111]. PCR efficiency, R^2^ value and slope value for the three reference genes which fall within acceptable range [112] were tabulated in Additional file 5. All assays were performed in three individual technical replicates of samples and non-template control was included.

**Table 8:** Primers used for quantitative real-time PCR analysis

Primers name	Sense sequence (5′-3′)	Antisense sequence (5′-3′)
*EgGAPDH 2*	GAAGGTCATCATATCTGCTCCC	CATCAACAGTCTTCTGAGTGGC
*EgNADH 5*	GCTCCCCTTTATTTGAATACCC	AATAGTTAGAGATGCCGCAAGC
*Egβ-Actin*	GAGAGAGCGTGCTACTCATCTT	CGGAAGTGCTTCTGAGATCC
*EgPR-1*	GTCAGGCAGCTCAACTTCAC	TCGAACTTGAACTGGGTCGA
*EgPG*	CTGGAGTGAAGATTAGTCAGGTG	ACAGAACTAGAGGCAGTAACATG
*EgEXP18*	ATGGCTACTTCTCTCCTGGC	CTTGATCCACAGCATTGCGA
*EgMYC2*	CTCAATCAGAGATTCTACGCCC	CCTTGAGGGTATCAACTTGGC
*EgBGIA*	ATGCACTGGGAAGAGCTCAT	GATGCCATCTTTGTCCACCC
*EgCht1*	AGCTCATCACTGTTCGACCA	CAAGAAAGCAGCGATCTCCC
*EgFIT*	GTGAAGTTGGAGTGCAGCAA	TCGCTGTCATCTCGAACTCA
*EgMTP10*	TTGGCAGTTATCGCTTCCAC	TGCAGACCAAGTGTAGCCAT
*EgPIN8*	GGTGGTGCTCGTATTGTGTC	CGAACCCTCCATGATGCTTG
*EgERF113*	AGCAGCACTAAAGTTCAAAGGC	GAATAAGGTCTGGGTAGGAGGG

### Statistical analysis

According to RNA-seq data, DEGs were determined following cut off-values of log_2_ FC ≥ |1.0| and *P*-value < 0.01. The expression of each genes from qPCR analysis was normalized by three reference genes; *GAPDH 2, NADH 5* and *ß-actin* expression levels. Expression levels were expressed as the mean ± SEM of three individual technical replicates of each sample. *P* < 0.01 denoted significant different between groups as assessed by one-way ANOVA analysis followed by Tukey’s test.

## Additional files

Additional file 1: Alignment of *Ganoderma boninense* PER71 ITS1/2 sequence with sequenced amplicon of normal and nested PCR. Nested primers were generated from sequenced PCR product of 3 days post infected oil palm root sample. Result showed conserved sequence which confirmed that *G. boninense* fungal hyphae were present in all T1 samples. (JPG 235 kb)

Additional file 2: Top-hit species distribution of best-aligned gene annotations with highest percentage of similarity and lowest e-value. With restriction to 20 blast hits and e-value cut-off of 0.001, *Elaeis guineensis* was the most top-hit species with close to 9000 top-hits for upregulated genes and 15,000 top-hits for downregulated genes. (JPG 173 kb)

Additional file 3: Statistics for blast and annotation procedures generated by Blast2Go Pro package from upregulated genes. (A) Annotation distribution; (B) E-value distribution; (C) Sequence similarity distribution; (D) Number of sequence with length. (JPG 118 kb)

Additional file 4: Statistics for blast and annotation procedures generated by Blast2Go Pro package from downregulated genes. (E) Annotation distribution; (F) E-value distribution; (G) Sequence similarity distribution; (H) Number of sequence with length. (JPG 125 kb)

Additional file 5: PCR efficiency, R^2^ value and slope value for the three reference genes. (PPTX 38 kb)

## Abbreviations

AFB: Auxin signalling F-box protein
BSR: Basal stem rot
COBL-4: Cobra-like 4
CR-4: CRINKLY-4
CWDEs: Cell wall degrading enzymes
d.p.i: Days-post-inoculation
DAMP: Damage-associated molecular pattern
DEG: Differentially expressed genes
ERF: Ethylene-responsive transcription factor
ETI: Effector-triggered immunity
EXP: Expansin
FC: Fold change
FIT: Fer-like iron deficiency-induced transcription factor
FRO2: Ferric reduction oxidase 2
GLIP: GDSL esterase/ lipase
GO: Gene Ontology
GSEA: Gene set enrichment analysis
HR: Hypersensitive response
IAA: Indole-acetic acid
Ile: Isoleucine
ITS: Internal transcribed spacer
JA: Jasmonate
MGDG: Monogalactosyldiacylglycerol
MYC: Transcription factor MYC
NGS: Next-generation sequencing
PAMP: Pathogen-associated molecular pattern
PCD: Programmed cell death
PCR: Polymerase chain reaction
PER: Peroxidase
PI: Protease inhibitor
PID: Protein kinase PINOID
PIN: Auxin efflux carrier
PR: Pathogenesis-related protein
PRR: Pattern recognition receptor
PTI: Pattern-triggered immunity
RBOH: Respiratory burst oxidase homolog
RIN: RNA Integrity Number
RLKs: Receptor-like kinases
RNA-seq: RNA sequencing
RNS: Reactive nitrogen species
ROS: Reactive oxygen species
RWB: Rubber wood block
SAR: Systemic acquired resistance
SCF^TIR1/AFB^: SKP-Cullin-F box, transport inhibitor resistant1/ auxin signaling f-box
SCWs: Secondary cell walls
TIR: Transport inhibitor response
TPM: Transcript per kilobase million
