# Supplementary figures and images for: Transciptome profiling at early infection of Elaeis guineensis by Ganoderma boninense provides novel insights on fungal transition from biotrophic to necrotrophic phase
Source: BMC Plant Biol. 2018 Dec 29;18:377. doi: 10.1186/s12870-018-1594-9 (PMC6310985; doi:10.1186/s12870-018-1594-9)

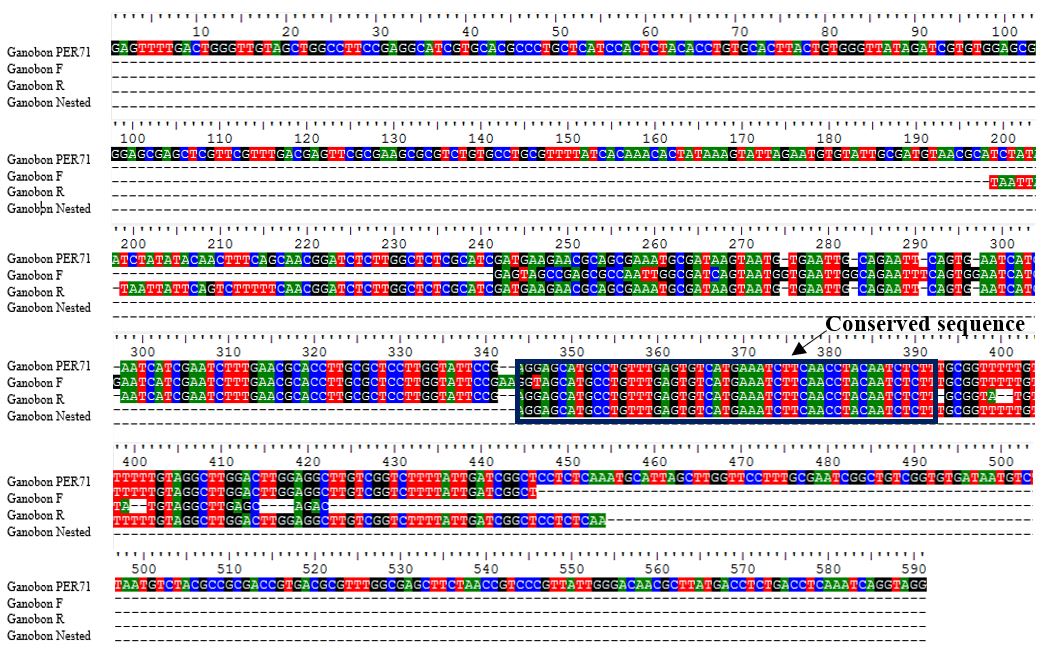

Supplement: Supplementary file 1 — Alignment of Ganoderma boninense PER71 ITS1/2 sequence with sequenced amplicon of normal and nested PCR. Nested primers were generated from sequenced PCR product of 3 days post infected oil palm root sample. Result showed conserved sequence which confirmed that G. boninense fungal hyphae were present in all T1 samples. (JPG 235 kb) [file 12870_2018_1594_MOESM1_ESM.jpg]

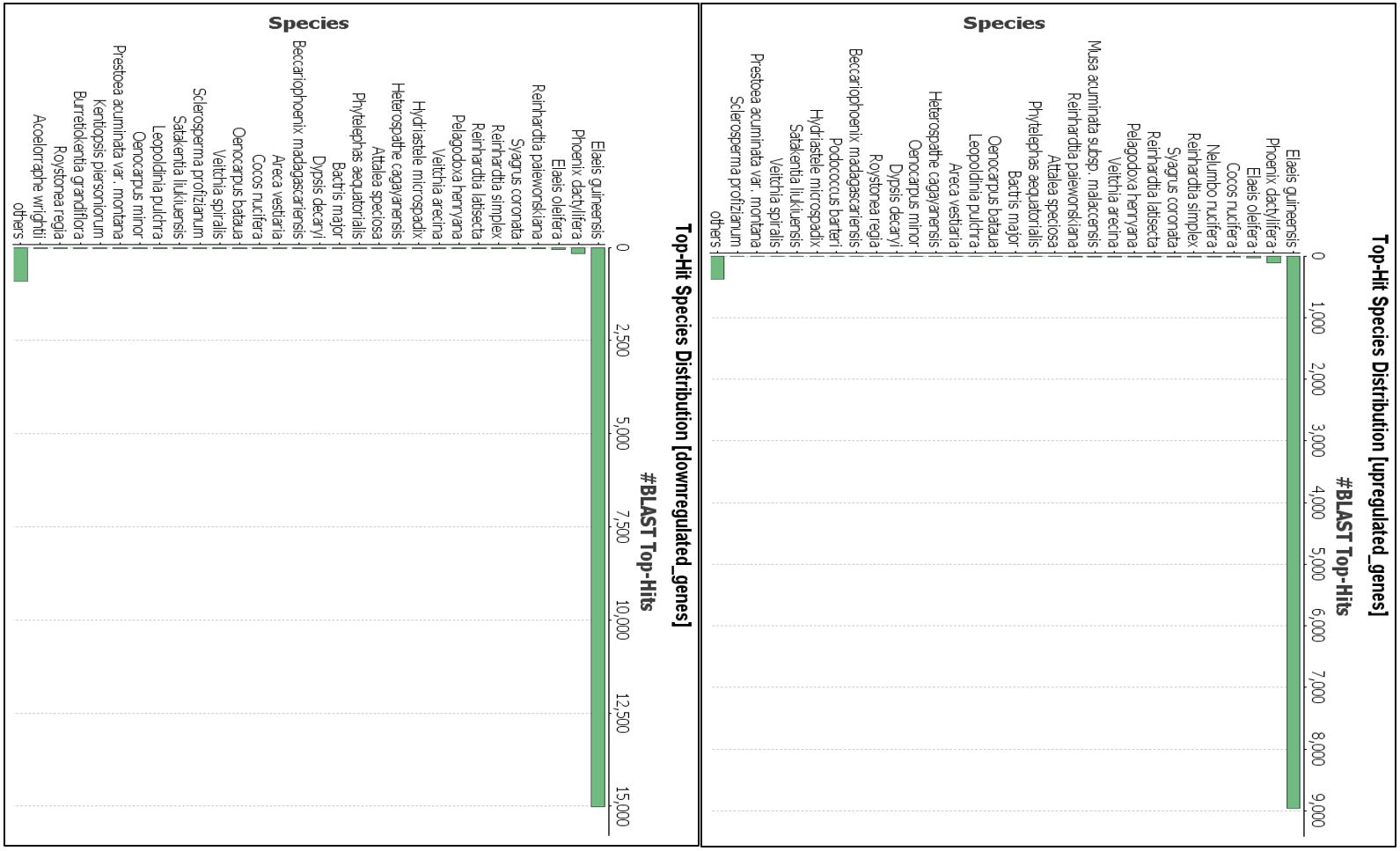

Supplement: Supplementary file 2 — Top-hit species distribution of best-aligned gene annotations with highest percentage of similarity and lowest e-value. With restriction to 20 blast hits and e-value cut-off of 0.001, Elaeis guineensis was the most top-hit species with close to 9000 top-hits for upregulated genes and 15,000 top-hits for downregulated genes. (JPG 173 kb) [file 12870_2018_1594_MOESM2_ESM.jpg]

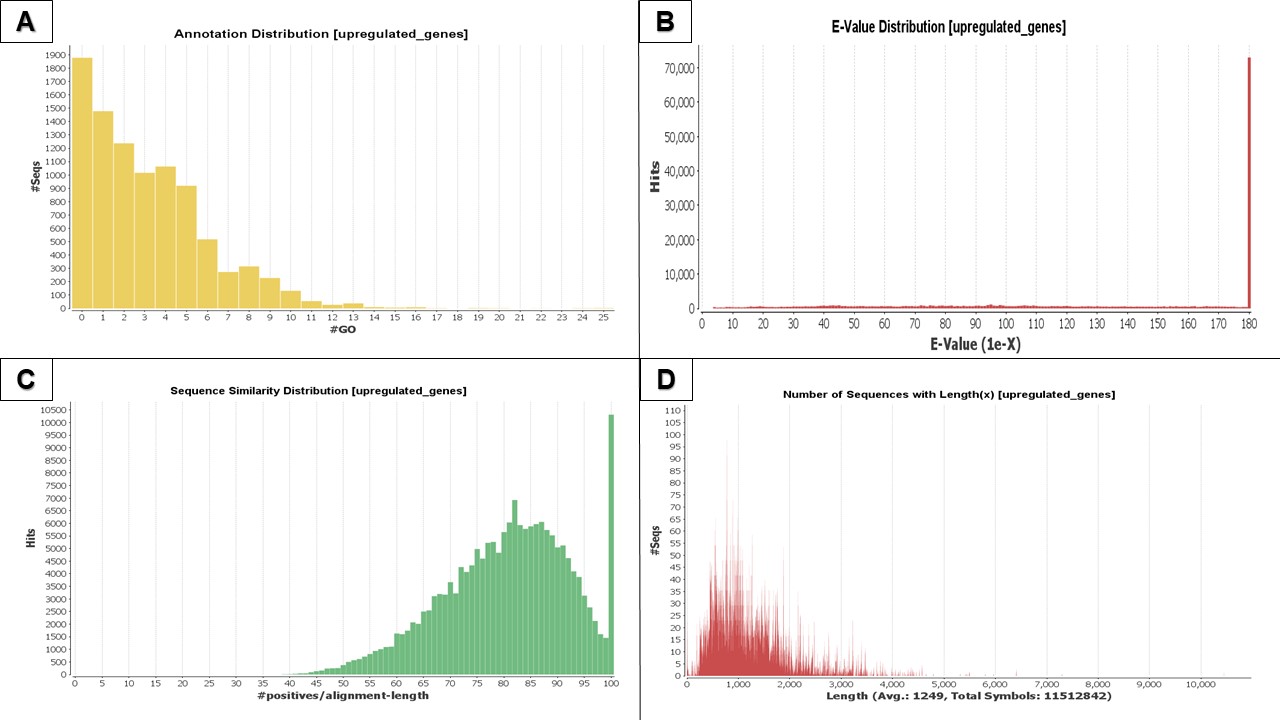

Supplement: Supplementary file 3 — Statistics for blast and annotation procedures generated by Blast2Go Pro package from upregulated genes. (A) Annotation distribution; (B) E-value distribution; (C) Sequence similarity distribution; (D) Number of sequence with length. (JPG 118 kb) [file 12870_2018_1594_MOESM3_ESM.jpg]

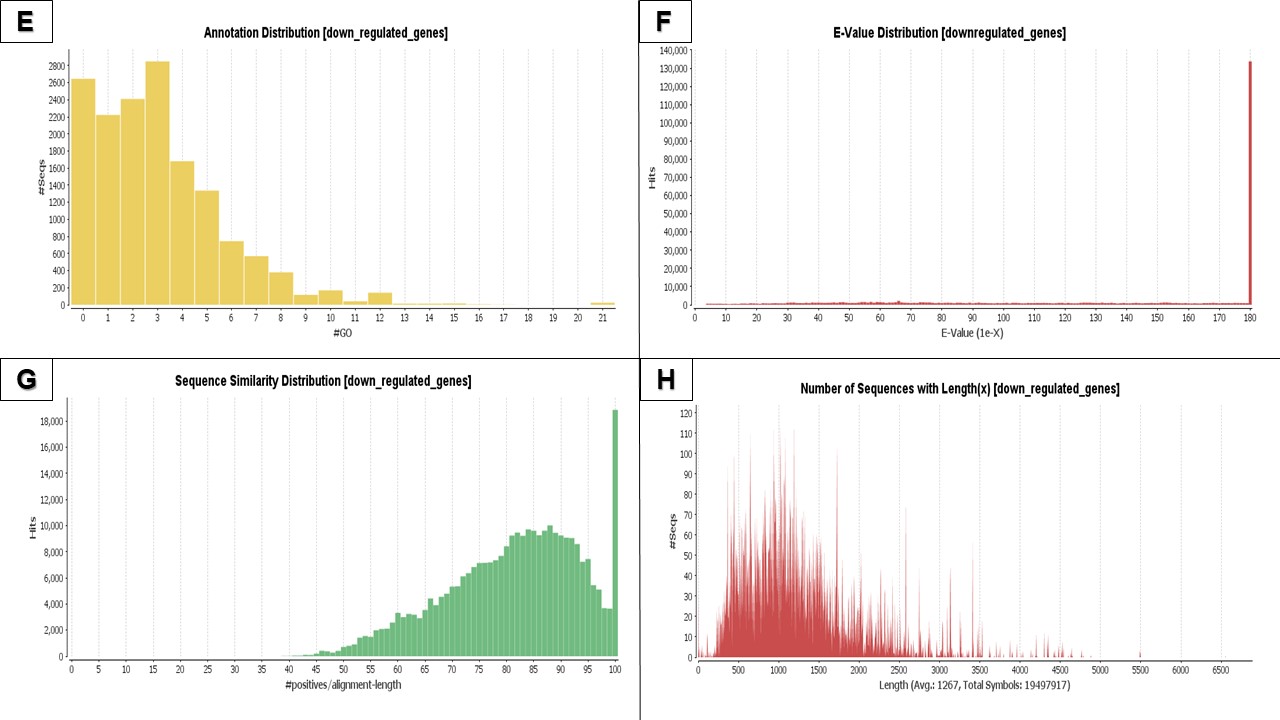

Supplement: Supplementary file 4 — Statistics for blast and annotation procedures generated by Blast2Go Pro package from downregulated genes. (E) Annotation distribution; (F) E-value distribution; (G) Sequence similarity distribution; (H) Number of sequence with length. (JPG 125 kb) [file 12870_2018_1594_MOESM4_ESM.jpg]
