# Supplementary material for: Transciptome profiling at early infection of Elaeis guineensis by Ganoderma boninense provides novel insights on fungal transition from biotrophic to necrotrophic phase
Source: BMC Plant Biol. 2018 Dec 29;18:377. doi: 10.1186/s12870-018-1594-9 (PMC6310985; doi:10.1186/s12870-018-1594-9)
Supplement: Supplementary file 5 — PCR efficiency, R2 value and slope value for the three reference genes. (PPTX 38 kb) [file 12870_2018_1594_MOESM5_ESM.pptx]

## Slide 1
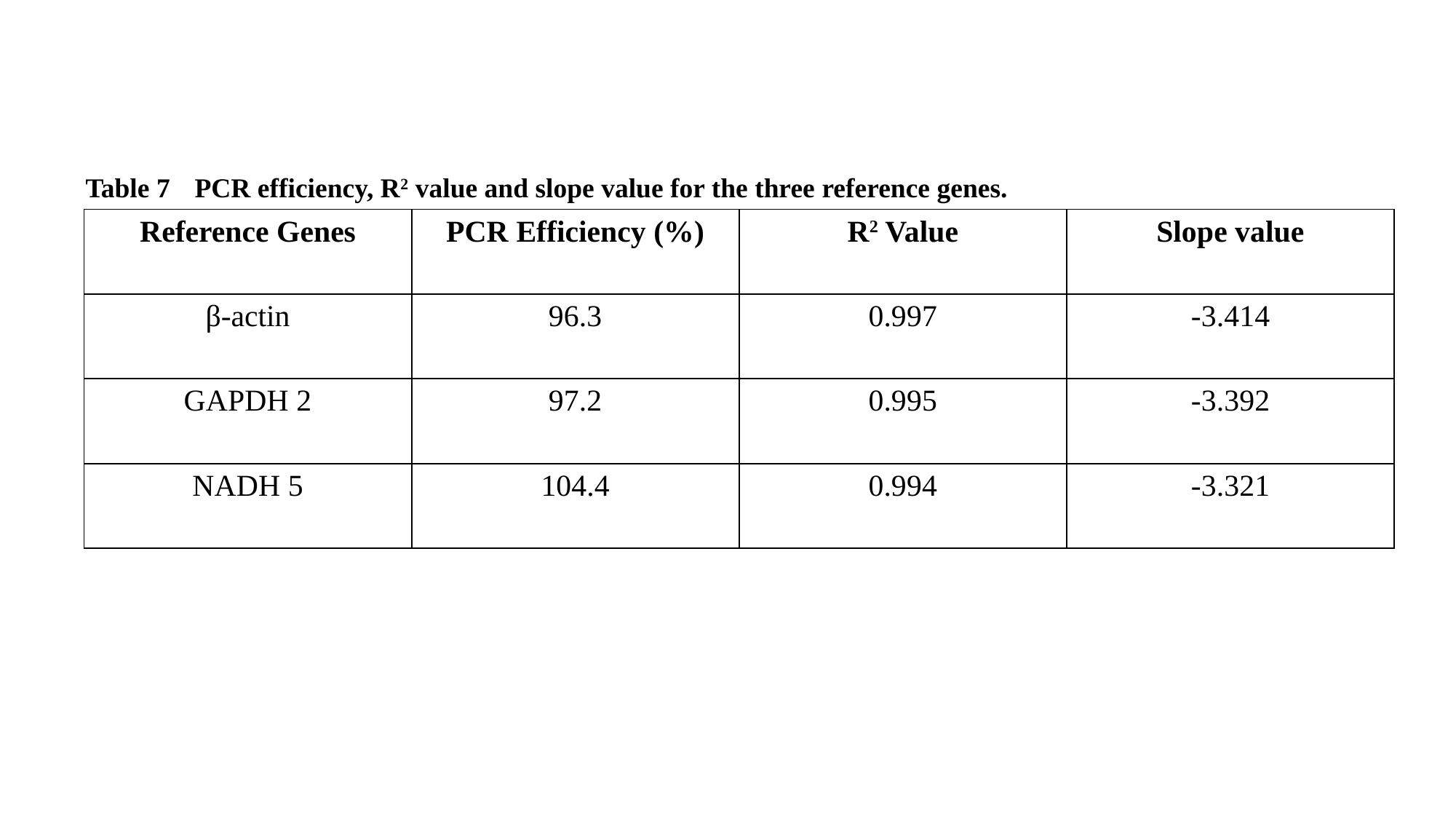

Table 7	PCR efficiency, R2 value and slope value for the three reference genes.
| Reference Genes | PCR Efficiency (%) | R2 Value | Slope value |
| --- | --- | --- | --- |
| β-actin | 96.3 | 0.997 | -3.414 |
| GAPDH 2 | 97.2 | 0.995 | -3.392 |
| NADH 5 | 104.4 | 0.994 | -3.321 |
